# Horizon-Dependent Solar Irradiance Forecasting with Boosted Trees, and Seasonal Baselines Based on Measurements in Sudan

**DOI:** 10.3390/s26185778

**Published:** 2026-09-11

**Authors:** Eltahir Idris Eltahir Mohamed, Devrim Akgun, Sohaib Ashri, Ahmad Khachan, Elfatih A. A. Elsheikh, Ceyda Aksoy Tirmikci

**Affiliations:** 1 Department of Information Systems and Technology, Sakarya University, Esentepe Campus, Sakarya 54050, Türkiye; 2 Department of Applied Physics and Mathematics, Faculty of Applied Sciences and Computer, Omdurman Ahlia University, Omdurman P.O. Box 786, Khartoum, Sudan; sohaibashri@gmail.com; 3 Department of Software Engineering, Sakarya University, Esentepe Campus, Sakarya 54050, Türkiye; dakgun@sakarya.edu.tr; 4 Sakarya University Research, Development, and Application Center (SARGEM), Sakarya University, Esentepe Campus, Sakarya 54050, Türkiye; 5 Department of Information Systems Engineering, Sakarya University, Esentepe Campus, Sakarya 54050, Türkiye; khachan.ahmad@gmail.com; 6 Department of Electrical Engineering, College of Engineering, King Khalid University, Abha 62521, Saudi Arabia; eelsheikh@kku.edu.sa; 7 Department of Electrical and Electronics Engineering, Sakarya University, Esentepe Campus, Sakarya 54050, Türkiye; caksoy@sakarya.edu.tr

**Keywords:** solar irradiance forecasting, multi-horizon forecasting, gradient boosting, ensemble learning, seasonal persistence, expanding-window validation

## Abstract

Solar irradiance forecasting accuracy depends on the prediction horizon because the use of recent observations, meteorological variables, and seasonal patterns changes with lead time. However, weak persistence baselines, temporally unreliable validation, and inconsistent test samples can overstate the advantage of complex models. This paper presents a leakage-safe, horizon-specific solar irradiance forecasting framework combining boosted trees, validation-weighted convex forecast combinations, and seasonal reference models. Direct forecasts are evaluated at 10-min and 1-, 3-, 6-, 12-, and 24-h horizons. Models are tuned using expanding-window validation; preprocessing is fitted only to the training data; and every method is evaluated on a predefined canonical test support. XGBoost achieves the lowest root mean square error (RMSE), 10.78 W/m^2^, at 10 min, whereas CatBoost achieves the lowest RMSE, 16.08 W/m^2^, at 1 h. At 3, 12, and 24 h, the lowest RMSE is obtained by seasonally guided forecast combinations. At 6 h, the XGBoost and two-day seasonal-mean combination ranks first by RMSE, although its gain over the two-day seasonal mean results in a larger mean absolute error (MAE). Daily-block significance tests show an improvement over the reference models at 10 min, while improvements over seasonal references at longer horizons are not statistically significant after adjustment. The framework provides a reproducible benchmark for horizon-dependent solar irradiance forecasting.

## 1. Introduction

The rapid growth of photovoltaic (PV) generation has increased the operational importance of accurate solar irradiance forecasting. Irradiance is the main atmospheric driver of PV, and errors in its prediction propagate to PV production forecasts, battery charging and discharging schedules, reserve acquisition, grid balancing decisions, and short-term market positions. Therefore, good forecasts can improve the scheduling of dispatchable resources, reduce unnecessary reserve margins, support storage operation, and limit the economic and reliability consequences of unexpected PV ramps [1,2,3,4,5]. These benefits are relevant at various decision levels. Very short-term forecasts support real-time ramp control and management. Forecasts from tens of minutes to several hours help with intraday dispatch, battery scheduling, reserve allocation, and market participation; and daily forecasts increasingly rely on numerical weather prediction and spatial atmospheric information [1,5,6,7]. Photovoltaic (PV) generation has become an increasingly important component of the transition to low-carbon electricity systems. Solar PV can provide electricity without direct fuel consumption or operational greenhouse gas emissions, and its modular nature allows deployment ranging from distributed rooftop installations to large-scale plants. However, photovoltaic production is intrinsically dependent on climate, since incident solar irradiance is the primary atmospheric factor determining the electrical energy available from a photovoltaic system. Therefore, the variability caused by cloud formation and movement introduces uncertainty in short-term PV production and poses challenges for the reliable and economical operation of the power grid [8,9]. The operational importance of forecasting is increasing as photovoltaic (PV) systems are integrated with other flexible and renewable sources. Irradiance and PV forecasts can support real-time compensation, economic deployment, reserve allocation, short-term market participation, and planning of energy storage systems [9,10,11]. In microgrids, these requirements are particularly important when intermittent PV and wind power generation operate alongside battery storage and vehicle-to-grid (V2G) sources. Recent studies have shown that coordinated control of PV, wind, storage, loads, and V2G-enabled electric vehicles can improve the reliability and economic performance of microgrid energy management [12]. More generally, electric vehicles can serve not only as electric loads but also as distributed, resilient resources that participate in electricity markets and provide grid support services through V2G operation [13]. Therefore, forecast errors are significant beyond their statistical magnitude. Errors in expected solar energy production can cause deviations between planned and actual production, alter balance and reserve requirements, affect exposure to electricity market imbalances, and change charging or discharging decisions for storage resources. A comprehensive review of the value of solar energy forecasting shows that the operational and economic consequences of forecasting errors largely depend on the application, market structure, decision-making process, and use of forecasting [10]. Similarly, the development of more manageable PV systems is increasingly integrating forecasting, energy storage, and curtailment to manage the uncertainty and variability of solar energy production [11].

This study focuses on the forecasting component of the operational chain. It evaluates global solar irradiance in physical units of W/m^2^ and does not simulate a specific PV plant, battery, reserve market, or energy management controller. Therefore, an improvement in RMSE or MAE irradiance should not be directly interpreted as an equivalent reduction in operating costs, reserve capacity, or battery utilization. Such an interpretation would require additional system-specific information, including installed PV capacity, module and inverter specifications, storage limitations, demand profiles, market prices, reserve regulations, and operational objectives. However, improving the accuracy and reliability of irradiance input provides a necessary foundation for these downstream photovoltaic and energy management system applications. The forecast horizon significantly influences a model’s predictive capacity and the information available for its use. Within a 10-min forecast horizon, the most recent irradiance observation is very valuable due to the marked short-term continuity of cloud fields and the resulting surface irradiance. When the horizon extends to 1 h, recent meteorological and irradiance measurements, together with temporal characteristics and nonlinear interactions, continue to provide significant predictive information. However, local cloud formation and advection introduce greater uncertainty. In situations with horizons of 3 to 6 h, the relevance of the most recent local measurement begins to decrease, while the importance of daily recurrence patterns becomes more pronounced. The challenges encountered at 12- and 24-h time horizons are similar to those faced in extended intraday and day-ahead forecasts, where single-station measurements often fail to predict the evolution of cloud fields. In these cases, using multi-day seasonal analogs may be a more competitive option. Numerous reviews show that the effectiveness of historical measurements, satellite imagery, local meteorological data, numerical weather predictions, and ensemble methods varies with time horizon [2,4,5,6,14]. It is crucial to recognize that a forecasting method effective at 10 min does not necessarily maintain the same level of effectiveness over longer periods, such as 1, 3, 6, 12, or 24 h.

Despite advances in machine learning for solar forecasting, methodological choices can complicate the interpretation of reported benefits. Random training and testing partitions can introduce future meteorological and seasonal information into model development, leading to optimistic estimates of operational performance. Time series evaluation must preserve temporal order, particularly in the presence of non-stationarity and distribution drift [15,16]. Comparisons based on ordinary persistence may exaggerate the apparent value of complex models at longer horizons. Persistence progressively weakens as lead time increases, while climatology, daily persistence, smart persistence, and seasonal reference combinations can remain highly competitive [17,18,19]. Sometimes models are evaluated on different subsets after model-specific filtering or sequencing, so reported errors do not necessarily correspond to the same forecast origins. Target leakage can enter through incorrectly shifted variables, target timing measurements, duplicate sensors, or undocumented channels that effectively reproduce the irradiance target. A single stochastic training run does not describe the variability of neural networks, multilayer perceptrons, or ensembles of random trees. Small numerical differences are often interpreted as evidence of model superiority without paired statistical analysis or correction for multiple comparisons.

These considerations are also important when studying forecast combinations. Equal-weighted averages and convex-weighted averages can reduce error when member forecasts are complementary, but offer little benefit when member residuals are strongly correlated. Therefore, a reliable time series ensemble should select its members and weights using chronologically generated expanding-window out-of-fold predictions, rather than the final test set. Non-negative simplex constraints are useful in this context because they preserve interpretability, avoid extreme extrapolation, and allow us to determine whether an apparent ensemble gain reflects a genuine combination of complementary forecasts or simply a quasi-degenerate dependence on one member.

This study develops a reproducible benchmark for predicting horizon-dependent solar irradiance at 10-min and 1-, 3-, 6-, 12-, and 24-h intervals. The timestamp audit constructs the complete 10-min grid and represents the three missing days as explicit missing rows. These gaps are not interpolated or treated as observed data. Forecast comparison includes robust seasonal baselines, individual learning models based on trees and tabular methods, and seasonal combinations of trees weighted by convex validation. Each model is evaluated on the canonical test support, a pre-declared and model-independent set of forecast origins, preventing differences in valid sample support from influencing the ranking. The reported experiment uses a fixed and reproducible seed. It is evaluated using full and daylight-only timestamp metrics, along with paired daily block significance tests followed by a Holm correction [20].

### 1.1. Related Work

Solar irradiance forecasting has generated great interest due to its relevance for the generation and operation of photovoltaic energy. The prediction horizon, data availability, baseline selection, underlying physical processes, useful input data, and operational applications vary depending on the prediction horizon, persistence, and seasonal baseline, as well as with machine learning, ensemble prediction, and the research gap addressed in this study. Solar forecasting methods are commonly distinguished by their lead time, as the underlying physical processes, useful input data, and operational applications vary with the prediction horizon. While the terminology is not completely standardized, very short-term or nowcasting problems generally range from a few minutes to about an hour, short-term and intraday forecasting range from tens of minutes to several hours, and day-ahead forecasting addresses the next operating day [1,2,3,6]. Very short-term forecasts are closely related to local cloud motion and recent irradiance dynamics, making high-frequency ground measurements and sky images particularly valuable [4,21,22]. At longer intraday horizons, the problem increasingly reflects the evolution and advection of cloud fields, and spatial observations, satellite information or numerical weather predictions may become more important [2,7,14].

The six horizons used in this work span different forecast regimes. The 10-min horizon represents a very short-term operational horizon, where persistence and recent target lags are difficult to overcome. The 1-h horizon is close to the boundary between the local short-term forecast and the intraday forecast. The 3- and 6-h horizons are operationally relevant for storage scheduling, intraday market updates, reserve planning, and short-term dispatch, but are also more challenging for a sensor-only local system. The 12-h horizon represents an extended intraday forecast, while the 24-h horizon approximates day-ahead operation and places greater emphasis on the recurring daily structure. The 2026 skill score meta-analysis by Nguyen and Müsgens [5] provides quantitative evidence that the forecast horizon has a central influence on model performance and that the utility of input categories changes between hourly, intraday, and next-day forecasting. The recent systematic review by Ghodusinejad et al. [6] similarly organizes physical, satellite, and AI approaches by forecast horizons. These findings support evaluating each forecast horizon separately, rather than averaging them or assuming a universally optimal architecture.

Recent work has incorporated increasingly rich data sources to expand useful forecast horizons. Niu et al. [14] combined public camera images with time series inputs in a multimodal transformer for intraday irradiance forecasting, while the deep learning review by Wang et al. [7] emphasizes multimodal learning, reliability, and interpretability in short-term solar forecasting. These systems can provide information on how to approach cloud fields, information that is not available in a single-station tabular dataset. This article addresses a complementary question: to what extent can a carefully audited record of local sensors, engineered temporal features, boosted trees, seasonal references, and leakage-safe ensembles be maintained before additional atmospheric information is required?

Persistence is the most used deterministic reference in solar forecasting. For a forecast issued at time *t*, conventional persistence assumes that the most recently observed irradiance remains constant over the entire forecast horizon. This assumption is demanding at very short horizons, as the irradiance exhibits high autocorrelation between adjacent samples, but deteriorates rapidly as the prediction horizon increases. Consequently, the choice of the reference model significantly affects the accuracy of the results and the apparent improvement attributed to a proposed method [5,17,18].

Several alternatives provide stronger references that better fit the prediction horizon. Smart persistence applies persistence to a clear-sky or clarity index and reconstructs future irradiance using a clear-sky model. Daily or seasonal persistence predicts irradiance at the target time using observations from the same time of the previous day. Multi-day means and medians reduce sensitivity to abnormal prior days by adding multiple daily analogs. Climatology and optimal convex combinations of climatology and persistence provide additional reference points whose suitability depends on the autocorrelation of the lag and the forecast horizon [17,18]. Analogous recommendations for constructing benchmark forecasts have been made in the probabilistic setting [23]. The analysis in Voyant et al. [18] demonstrates that the most appropriate naive benchmark depends on seasonality, cyclicality, forecast horizon, and the selected error measure. Therefore, showing improvement over ordinary persistence alone does not establish that a learning model adds useful information.

Clear-sky normalization is common because it largely removes the deterministic solar cycle, allowing models to focus on atmospheric dimming. However, it introduces dependency on the selected clear-sky model, site metadata, aerosol assumptions, and temporal alignment. The Voyant et al. [19] study demonstrated that clear-sky-based short-term prediction can be sensitive to such assumptions and investigated a non-clear-sky alternative based directly on global horizontal irradiance. Therefore, for a dataset whose exact geographic metadata is unavailable or has not been independently verified, an observed daily analog may be a more reliable primary baseline than a clear-sky method based on uncertain coordinates. The present comparative analysis includes ordinary persistence, one-day seasonal persistence, a two-day seasonal mean, and a seven-day seasonal median. It also evaluates predictive accuracy relative to seasonal benchmarks, rather than relying only on ordinary persistence. This design responds to recent calls for more robust and transparent benchmarks for solar forecasting [5,17,18,24]. It is particularly important for 3- and 6-h horizons, where daily recurrence may exceed the predictive information contained in the latest local meteorological measurements.

Machine learning models are attractive for solar irradiance forecasting because they can represent nonlinear relationships between recent irradiance, meteorological measurements, calendar variables, lagged observations, and rolling statistics without requiring an explicit atmospheric model. Reviews have documented the widespread use of neural networks, support vector regression, regression trees, random forests, and boosting methods in irradiance and photovoltaic prediction [3,6,25]. Recent comparative studies continue to report robust results for boosted tree algorithms in different climates and input configurations [26].

Random Forest builds an ensemble of uncorrelated trees using bootstrap samples and random feature subsets [27]. Extra Trees increases randomization by selecting split thresholds more randomly and can reduce variance with low computational cost [28]. Instead, gradient boosting builds trees sequentially so that each learner corrects errors made by the previous set [29]. XGBoost extends this framework with regularized objectives, shrinkage, column subsampling, and efficient split construction [30]. LightGBM uses histogram-based learning and leaf-wise tree growth to improve scalability [31], while CatBoost was designed to reduce prediction shift and handle categorical variables using ordered boosting [32]. Even when the input variables are predominantly numerical, alternative tree-growth and regularization strategies from these libraries can yield distinct residual patterns, thereby providing useful ensemble diversity.

Boosted trees are particularly suitable for the current environment. The dataset is of moderate size, the predictors are structured rather than image-based, and much of the temporal information can be expressed through explicit lag, rolling, seasonality, and calendar features. Tree models require little scaling, accommodate nonlinear interactions, and can capture horizon-specific thresholds and climate regimes. At the same time, related boosting implementations trained on the same features can produce highly correlated forecasts. Their individual similarity is a reason to evaluate forecast combinations using out-of-fold validation rather than assuming that combining more models is inherently beneficial. Linear Ridge regression and a multilayer perceptron are retained as secondary comparators. Ridge regression reduces the coefficients and remains useful when the predictors are collinear [33]. Multilayer feedforward networks are flexible nonlinear approximators [34], but their performance can be sensitive to scaling, architecture, optimization, and random initialization. In a single-year, single-site dataset, more flexible models can overfit overlapping observations even when the number of windows generated appears large.

Deep sequence architectures have become prominent in solar forecasting because they learn temporal representations directly from historical windows. Long short-term memory (LSTM) networks use gated memory to model long dependencies [35], gated recurrent units (GRUs) offer a simpler recurrent alternative [36], and transformers use self-attention to model long-range relationships [37]. Solar-specific reviews describe rapid growth in multimodal, recurrent, convolutional, and attention-based models [4,7]. However, greater architectural sophistication does not guarantee superiority across all datasets or horizons. The effective number of independent atmospheric situations can be much smaller than the number of overlapping windows, and local time series cannot directly observe future cloud fields. Deep sequence architectures are not included in the present experimental comparison and remain an important extension for future multi-year, multi-site studies. Ensemble forecasting seeks to exploit complementary errors between individual methods. The simplest regression ensemble is an arithmetic mean. A convex weighted average generalizes this approach by learning non-negative weights that sum to one. In classification, probability averaging is often referred to as a soft vote. For continuous irradiance forecasting, the most accurate terminology is weighted average or regression voting. Convex constraints improve interpretability and prevent extreme extrapolation, but the resulting combination cannot correct a shared bias if all members fail in the same direction.

Solar forecasting studies have reported the benefits of combining statistical, machine learning, image-based, and physical predictions. Hategan et al. [22] combined statistical extrapolation, gradient boosted trees, LSTM, and sky imagery for intra-hour forecasts and reported that a weighted combination outperformed a simple average on RMSE-based skill, although the simple average retained an advantage on bias and percentage error. Shan et al. [38] proposed a multimodal intra-hourly ensemble using heterogeneous inputs, while Carneiro et al. [39] used regularized regression to combine machine learning forecasts for solar and wind applications. These studies indicate that ensemble gains are more likely when members encode genuinely different information sources or modeling assumptions. The present study uses combinations of convex forecasts, with weights selected exclusively from expanding-window out-of-fold predictions. This design prevents the final testing period from influencing the combination and allows the learned weights to be directly interpreted. It is especially relevant to combine boosted tree forecasts with daily seasonal analogs because these members represent different prediction mechanisms: nonlinear correction of recent sensor conditions and recurrence at the same target clock time on previous days. Reliable model comparison requires more than selecting an error metric. Time-dependent observations violate the assumption of independence and identical distribution behind random validation. Bergmeir et al. [15] studied the conditions under which cross-validation can be valid for autoregressive prediction, while the extensive empirical analysis of Cerqueira et al. [16] found that cross-validation variants performed competitively on stationary synthetic series but that temporally ordered out-of-sample procedures were more reliable on real-world series. Solar irradiance is strongly seasonal and meteorologically non-stationary; Consequently, expanding-window validation provides a closer approximation to deployment than random folds.

Support for evaluation is another source of sample-selection bias. Sequence models may lose samples due to lookback requirements, missing intervals, or unavailable seasonal analogs, while tabular methods may retain them. If each model is evaluated on its own valid subset, differences in error may partly reflect differences in sample difficulty. The current study avoids this problem by defining canonical test support independently of the final model list and then requiring that each reported model predict the same forecast origins. Standard metrics like RMSE and MAE are still useful because they measure errors in physical units, but RMSE emphasizes large deviations, while MAE is more robust to extreme events. Skill scores add a relative perspective when comparing a model to a specific reference. Its interpretation only makes sense when the baseline is explicitly defined and sufficiently robust [5,17]. Recent work has also proposed multidimensional measures to improve the discrimination and interpretability of deterministic solar forecasts [24]. In the present study, RMSE, MAE, $R^{2}$, normalized errors, biases, and skill scores are complemented by analyses of ramp, monthly, hourly, irradiance, and daylight-only event conditions. Small differences in performance should be tested using paired errors calculated from common observations. Because adjacent 10-min errors are serially dependent, this work aggregates daily absolute errors before comparing the methods. Both paired *t* tests and Wilcoxon signed-rank tests are reported, and Holm’s sequential procedure controls for the familywise error rate in related model comparisons [20]. The reported experiment uses a fixed reproducible seed. Repeated evaluation of seeds is still necessary before making claims about stochastic stability between runs.

### 1.2. Research Gap

The reviewed literature demonstrates advances in solar irradiance and PV forecasting using statistical models, machine learning methods, deep learning architectures, hybrid approaches, and ensemble forecasting. However, the comparison of forecasting methods can be strongly affected by the experimental design. In particular, weak baseline forecasts, random temporal partitioning, inconsistent test samples, preprocessing outside the training period, unidentified target-proxy variables, and forecast combinations adjusted with information from the final test period can yield optimistic or mismatched results. A second limitation refers to the forecast horizon. The predictive value of recent irradiance observations, meteorological measurements, and seasonal information varies with lead time. Consequently, the conclusions obtained for a very short-term forecast cannot be automatically generalized to intraday or daily conditions. Robust daily and multi-day seasonal references are especially important because a complex learning model should demonstrate improvement not only over ordinary persistence but also over forecasts that explicitly exploit the pronounced diurnal recurrence of solar irradiance. Therefore, there is a need for a common horizon-dependent evaluation framework that jointly addresses temporal data integrity, the availability of forecast-origin and target-time features, target-proxy exclusion, chronological model selection, robust seasonal baselines, identical testing support across competing models, and leakage-safe forecast combination. The present study addresses this gap by evaluating competing methods under a unified chronological protocol over forecast horizons ranging from 10 min to 24 h.

### 1.3. Contributions

This study makes several methodological and empirical contributions to forecasting solar irradiance across multiple horizons. The methodological contribution lies in integrating these elements into a common horizon-dependent benchmarking framework that simultaneously addresses multiple sources of optimistic or unfair comparisons. First, it introduces a reproducible and leakage-safe benchmarking framework for horizon-dependent forecasting that integrates dataset auditing, training-only preprocessing, expanding-window validation, and a common canonical test support to ensure fair and unbiased model evaluation. These procedures reduce optimistic performance estimates arising from temporal leakage, target-proxy information, inconsistent evaluation samples, and improper handling of missing data. Second, the study provides a comprehensive comparison among machine learning algorithms, seasonal baselines, and weighted combinations of forecasts across horizons ranging from 10 min to 24 h. In doing so, it highlights how forecast timing affects the importance of historical information, weather data, and seasonal patterns for forecast accuracy, offering guidance for model selection based on the forecast horizon. Third, the study proposes a forecast combination methodology that avoids leakage and uses only forecasts generated in chronological order to estimate ensemble weights. In the forecast-combination procedure, measures against degenerate, duplicate, and single-model solutions are used. Finally, the test can distinguish between good numerical performance and statistical improvement by evaluating all forecasting approaches over the same forecast horizon and using daily-block paired tests with Holm correction.

The contributions of this work are outlined as follows:

- A leakage-safe multi-horizon benchmarking framework that combines timestamp regularization, sensor-data auditing, target-proxy exclusion, gap-aware temporal processing, training-only preprocessing, and chronological model selection.
- A systematic comparison of machine-learning models with one-day and multi-day seasonal references over six forecast horizons ranging from 10 min to 24 h.
- Expanding-window out-of-fold model and ensemble selection followed by refitting on all valid pre-test observations and final evaluation on a canonical test support.
- Leakage-safe forecast-combination strategies, including validation-derived convex combinations based exclusively on out-of-fold predictions.
- A statistical evaluation based on daily error blocks and multiplicity-adjusted paired tests that distinguishes numerical improvements from statistically supported improvements.
- Empirical evidence that the usefulness of model complexity changes with forecast horizon, with nonlinear tree models being competitive at short horizons, while daily seasonal information becomes important at longer horizons.

## 2. Dataset, Audit, and Feature Preparation

This part focuses on how data collection and processing methods affect forecast validity. It is important to note that the goal of the data audit was not to eliminate anomalous data points but to reveal differences in timestamps, missing intervals, duplicate sensor channels, proxy target variables, and feature groups that could lead to data leaks and forecast misinterpretation.

### 2.1. Data Collection

**Figure 1:**
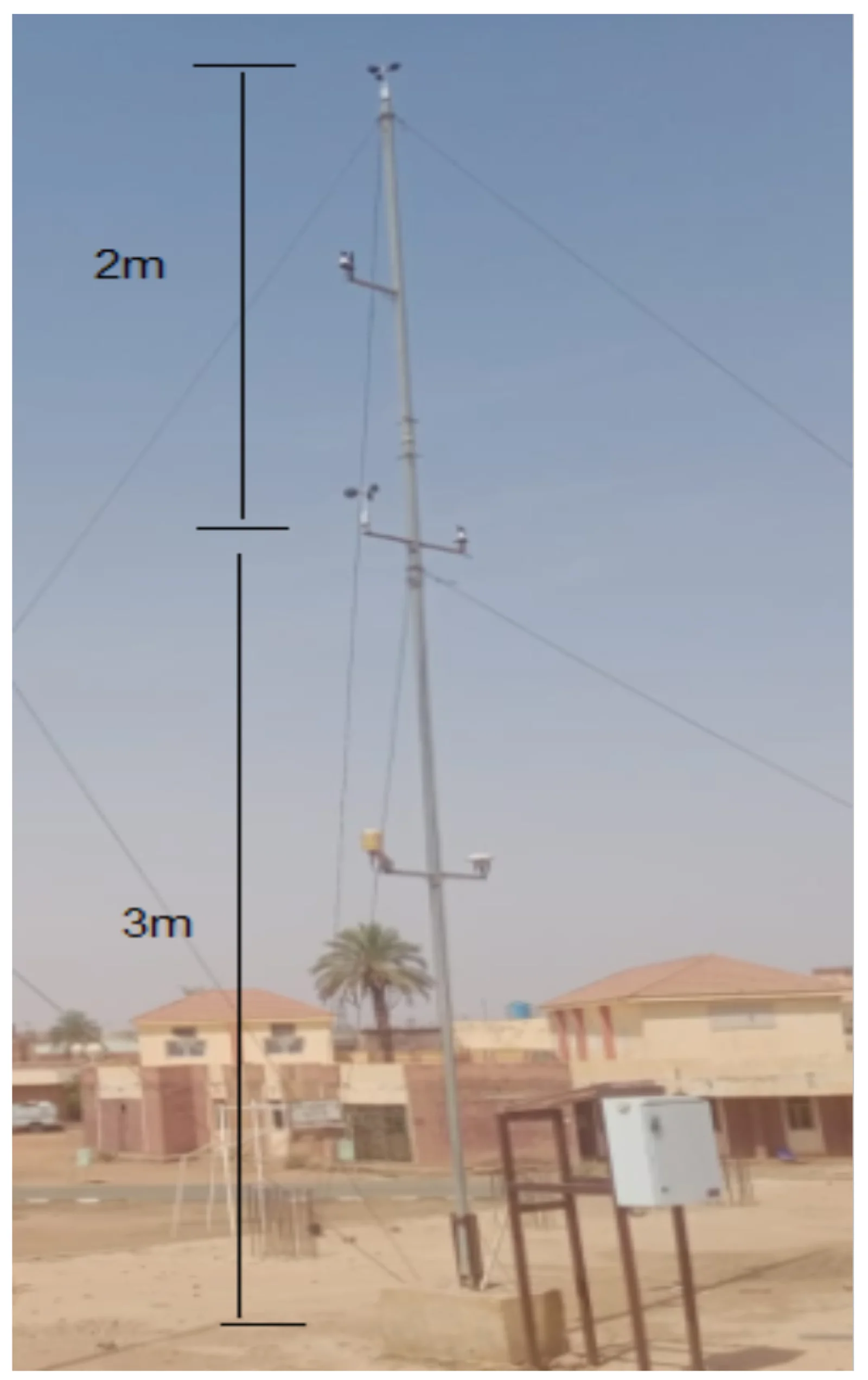
Meteorological station established on the rooftop of the Faculty of Engineering, Omdurman Ahlia University, Sudan.

The experiments were conducted between January and December 2018 at the meteorological station installed on the roof of the Faculty of Engineering, Omdurman Ahlia University, Sudan (15.63° N, 32.46° E). These coordinates are reported as descriptive station-location information. This station is located in an open area with minimal obstructions to wind flow and shade, making it ideal for meteorological measurements, as shown in Figure 1.

The weather station was equipped with sensors for global solar irradiance, wind speed and direction, air temperature, relative humidity, and atmospheric pressure. Global solar irradiance was measured using an MS-410 pyranometer that meets the requirements of ISO 9060 [40]. The pyranometer measures global solar irradiance in W/m^2^ and serves as the target variable for the forecasting experiments. Irradiance measurements were recorded as aggregated 10-min observations. Wind speed was measured using two Thies FCA X anemometers installed at measurement heights of approximately 3 m and 5 m above the station base. The reported operational uncertainty of the anemometers is approximately ±0.04 m/s with a wind speed of 10 m/s. Measurements at two heights provide information on variations in local wind conditions. Wind direction was measured using two first-class electronic vanes based on potentiometric sensors. The upper and lower measurements of wind speed and direction are stored as separate sensor channels in the dataset. Together, wind speed and direction provide information about local airflow conditions that can influence short-term meteorological variability.

Air temperature and relative humidity were measured using a combined temperature/humidity sensor unit. The temperature was measured using a PT100 platinum resistance temperature sensor with a measurement range of −40 °C to +80 °C and a reported accuracy of approximately ±0.3 °C. Relative humidity was measured using a thin-film capacitive sensing element over the range 0–100% RH, with a reported accuracy of approximately ±2–3% RH. These variables characterize the thermal and humidity state of the local atmosphere. Atmospheric pressure was measured using the station’s barometer, which provides an additional indicator of atmospheric conditions. The exact model designation of the barometer was not available in the station documentation and is therefore not specified.

**Figure 2:**
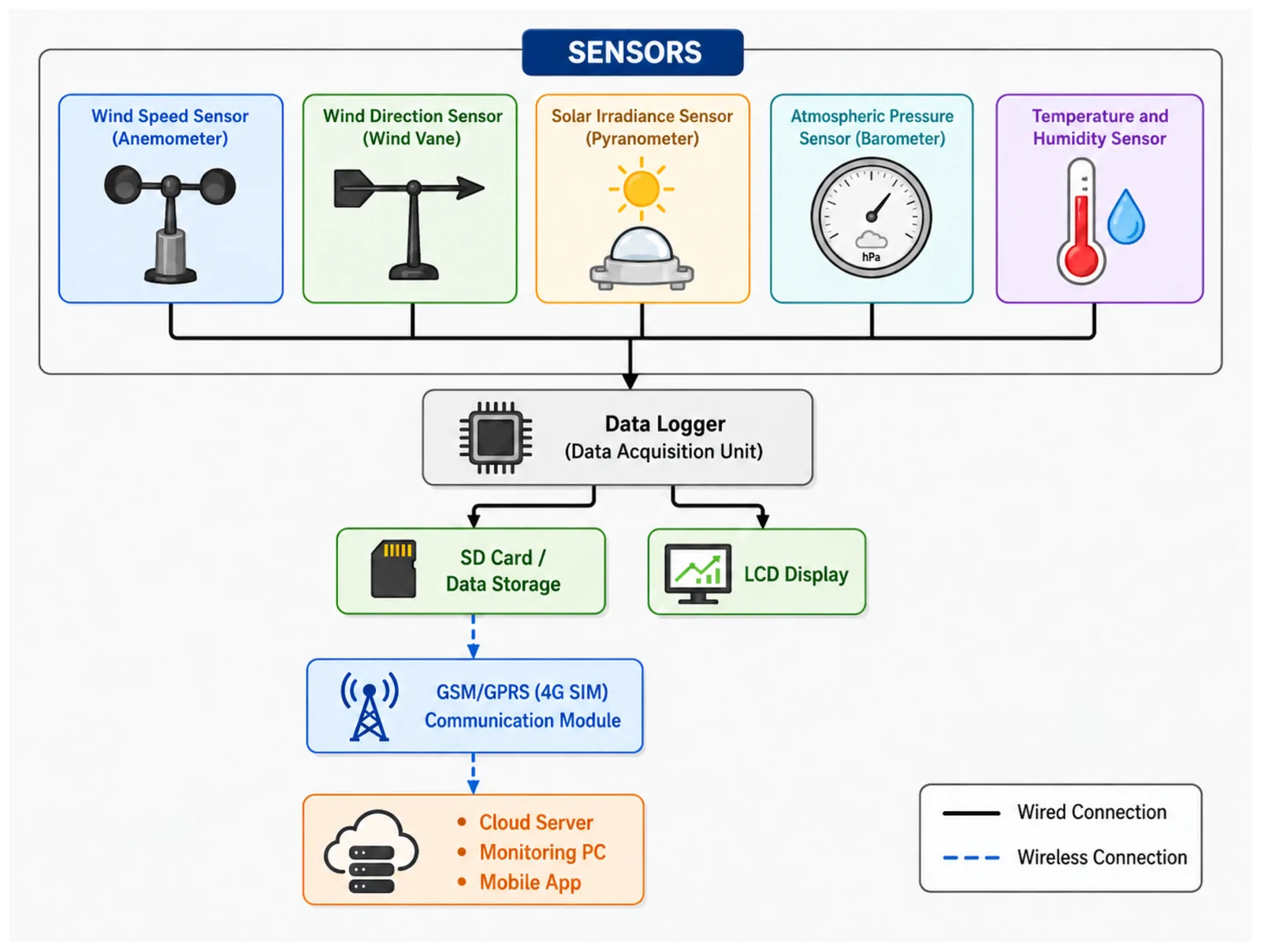
Measurement and data-acquisition architecture of the meteorological station. Wind speed, wind direction, solar irradiance, temperature, and relative humidity sensors provide measurements to the Meteo-40M Plus data logger.

Measurements were acquired using the Meteo-40M Plus data logger, which supports both analog and digital sensor inputs. As illustrated in Figure 2, sensors for wind speed and direction, solar irradiance, temperature, and relative humidity provide their measurements to the data logger. The logger records sensor measurements at 10-min intervals, producing 144 records per day and providing local storage and display capabilities. Archived measurements can also be transmitted via a GSM/GPRS/LTE communication module for remote monitoring and data access. Data collection was carried out throughout the year, from 1 January to 31 December 2018. The maximum solar irradiance recorded during the measurement period was 1058.48 W/m^2^, and the maximum air temperature measured in June was 45 °C. Since solar irradiance is not available at night, daylight-only performance was evaluated.

Figure 2 represents the measurement and data acquisition stage that precedes the forecasting procedure. Its purpose is to show how observations from physical sensors are collected, stored, and made available as a raw dataset. The forecasting methodology does not operate in the communication or visualization components themselves. Instead, it uses the 10-min logs produced by the data logger. These records serve as input for subsequent timestamp auditing, missing-interval analysis, sensor channel detection, feature preparation, chronological data splitting, model training, and forecast evaluation, as described in the following subsections.

### 2.2. Dataset Description

The experiments use measurements collected from January to December 2018 at a nominal 10-min sampling interval. The original raw data file contains 52,128 observed rows and 77 columns, including the timestamp field. The measured variables include wind speed, wind direction, relative humidity, air temperature, atmospheric pressure, solar irradiance, logger aggregation statistics, and various anonymous or electrical channels. The target of the forecast is the average global solar irradiance recorded during each 10-min interval. In this study, it is defined as

(1) $$G_{t}=\mathrm{Solar}\;\mathrm{Sensor};\mathrm{solar}\_\mathrm{irradiance};{\mathrm{Avg}}_{t},$$

where $G_{t}$ denotes the global solar irradiance measured at timestamp *t*, expressed in $W/m^{2}$. Equation (1) is the target definition used in all forecasting experiments.

The mentioned meteorological measurements are organized into several sensor families. Upper and lower anemometers record wind speed, while corresponding upper and lower vanes measure wind direction. Relative humidity and air temperature are measured with a combined hygrometer/thermometer, and atmospheric pressure is measured with a barometer. The solar sensor family provides average, minimum, maximum, and standard deviation statistics for irradiance at each 10-min interval. These named channels form the physically interpretable core of the dataset. In contrast, anonymous and electrical channels are examined separately during the audit because their meanings and availability at the expected time are less certain. The complete 2018 calendar at 10-min resolution contains 52,560 timestamps. Therefore, the dataset includes 52,128 observed timestamps and 432 missing grid positions. All observed timestamps are valid and unique, and no duplicate or malformed timestamps were detected. The temporal coverage and the resulting number of missing intervals are summarized in Table 1.

### 2.3. Timestamp Integrity and Temporal Gaps

The raw timestamp index was sorted first and examined for malformed, duplicate, and off-grid timestamps. A complete 10-min index was then constructed from the first to the last observation, and the original records were reindexed onto this regular grid so that each expected timestamp was explicitly represented. Consequently, a missing log record appears as a missing row rather than being silently skipped or hidden by irregular temporal spacing.

This regularization is important for both tabular and sequence-based forecasting models. It ensures that time lags correspond to fixed physical durations, unambiguously identifies missing intervals, and prevents a row lag from representing different elapsed times across different parts of the dataset. Off-grid timestamps are rejected by default unless an explicitly configured and independently audited snapping tolerance is applied.

The regularized time series contains 432 missing intervals, all of which occur in three complete one-day slices: 10 April 2018; 16 April 2018; and 15 May 2018. Each slice contains 144 10-min missing intervals and therefore corresponds to 24 h. Together, the three events represent 72 h of missing data and divide the annual record into four continuous time segments. This structure is preferable to a large number of isolated missing timestamps because the affected periods can be handled explicitly without introducing numerous local discontinuities. The missing days were not interpolated. Interpolation across a 24-h blackout would create synthetic irradiance and meteorological trajectories that are not supported by observations and would artificially smooth both the diurnal cycle and cloud-driven variability. Instead, missing timestamps remain explicitly marked in the regularized framework. For sequence models, a forecast origin is accepted only when each timestamp in its lookback window belongs to an observed continuous segment. Let $W_{t}\left(L\right)$ be the step history window *L* that ends at the origin *t*. A sequence sample is retained only when Equation (2) is satisfied.

(2) $$I_{\mathrm{valid}}\left(t,L\right)=\prod_{\tau \in W_{t}\left(L\right)}I_{\mathrm{observed}}\left(\tau \right)=1.$$

where $I_{\mathrm{observed}}\left(\tau \right)$ is equal to 1 if the timestamp τ is observed and 0 if it is missing. Therefore, $I_{\mathrm{valid}}\left(t,L\right)$ equals 1 only when all timestamps in the lookback window are available. This condition ensures that no history window crosses the boundary of a missing day. Gap membership is not masked by median imputation, because imputation is applied only to feature cells within otherwise valid time windows. The three identified interruptions occur during the training period, while the validation and final testing periods have none.

### 2.4. Sensor-Quality Assessment

The observed logger rows are internally consistent. In all original sensor cells, the audit identified no raw missing sensor values, infinite values, or failures during numerical conversion. No constant sensor columns or wide physical range violations were detected, and all reported standard deviation values were non-negative. The aggregation statistics were also internally consistent, with no violations of the expected relationship given by Equation (3)

(3) $$x_{t}^{\min}\leq x_{t}^{\mathrm{avg}}\leq x_{t}^{\max}.$$

Therefore, the 432 missing target values in the regularized framework are attributable to the absence of three full days, not to missing cells in the observed log records. The physical examination was used as a diagnostic tool rather than an automatic rule of thumb. In particular, irradiance observations near sunrise, sunset, and during cloudy periods were not removed because they appeared unusual under a fixed, clock-based definition of daylight. The raw data file contains eight logger channels Count. These variables are effectively duplicate measures of interval completeness and are nearly constant: 52,126 observed rows have a nominal count of 600, while 1 row has 583, and 1 has 582. Retaining all Count variables would introduce redundant predictors and could distort feature-importance analyses without providing meaningful prognostic information. In this way, the Count family was replaced by two consolidated quality indicators. Let $c_{t}$ be the minimum count value available across all accepted named sensor families at time *t*, and let $c^{*}$ be the expected count estimated from the training period. The generated variables are defined by

(4) $$q_{t}^{\mathrm{complete}}=I\;\left(c_{t}\geq c^{*}\right),$$

and

(5) $$q_{t}^{\mathrm{deficit}}=\max\;\left(0,c^{*}-c_{t}\right).$$

Equations (4) and (5) preserve the only potentially useful information in the Count channels, whether and to what extent an aggregation interval is incomplete, while avoiding eight nearly identical predictors. In the audited dataset, only two observed rows are marked as incomplete relative to the face value $c^{*}=600$.

### 2.5. Duplicate and Proxy-Channel Removal

The most important finding of the audit concerns undocumented channels that faithfully reproduce named sensor variables. Training-only correlation and linear fit diagnostics indicate that A1 approximately reproduces atmospheric pressure, A2 reproduces air temperature, A3 reproduces relative humidity, and A4 reproduces solar irradiance. Similarly, C1 and C2 approximately reproduce the wind speed measurements from the upper and lower anemometers, respectively. The A4;Avg channel is especially critical because its daylight correlation with the target irradiance is approximately 0.999998. The current measured irradiance $G_{t}$ is a valid predictor of $G_{t+h}$ when an irradiance history forecast profile is used. However, retaining both the named irradiance channel and A4 would essentially duplicate the same information. More importantly, a nominally meteorological-only experiment that excluded the named irradiance channel but retained A4 would silently reintroduce target history information and invalidate the intended interpretation of the feature profile.

Therefore, the main experiments exclude all channels A1–A4 and C1–C2. The undocumented D1–D2 families, electric or anonymous channels V, I, and T, logger identifier addr, and unrestricted raw count channels are also removed from the experiment’s default feature set. Electrical channels are not necessarily invalid measurements. Some are correlated with daylight irradiance and may contain useful operational information. However, their inclusion would change the scientific interpretation of the experiment, moving from a forecast based on meteorological and irradiance-history inputs to one based on a broader, multisensor power-system state. Therefore, these channels are reserved for separately declared supplemental ablation unless their physical definitions, measurement times, and real-time availability are independently verified.

### 2.6. Target Characteristics

The irradiance target exhibits the expected strong diurnal structure. Among the observed rows, approximately 48.76% of the target values are exactly zero, largely reflecting nighttime conditions. The overall mean is approximately 236.45 $W/m^{2}$, while the mean within the audit’s preliminary 06:00–18:00 clock window is approximately 472.47 $W/m^{2}$. The maximum observed irradiance is approximately 1058.48 $W/m^{2}$ and no negative target values were found. The preliminary clock-based daylight audit identified some positive irradiance outside of 06:00 to 18:00 and some low values within that range. These observations are consistent with seasonal variations in sunrise and sunset and cloud conditions and were not treated as sensor errors. When verified site coordinates and time zone information are available, solar elevation provides the preferred definition of physical daylight. In the absence of verified geographic metadata, daylight-only performance is evaluated conditionally using the observed target irradiance and is not used as a predictive input. The time dependence of the measured global solar irradiance was examined using lagged autocorrelation analysis. For each lag *k* selected, the measured irradiance values $G_{t}$ were matched to the corresponding lagged observations $G_{t-k}$. Equation (6) calculates the autocorrelation from valid pairs of the measured irradiance values $G_{t}$ and $G_{t-k}$ for a lag of *k* sampling intervals.

(6) $$\rho \left(k\right)=\mathrm{Corr}\left(G_{t},G_{t-k}\right),$$

where *k* is expressed in 10-min sampling intervals. The All Timestamps column in Table 2 uses all valid observation pairs. The daylight column was calculated separately from observations within the 06:00–18:00 local clock interval used in the preliminary descriptive analysis. The irradiance series was not seasonally adjusted before calculating these coefficients, because the purpose of the analysis was to identify the temporal structure naturally present in the measurements, including short-term persistence and daily and multi-day recurring patterns. With a lag of 10 min, the autocorrelation of all timestamps is approximately 0.994, and the preliminary value of the daylight subset is approximately 0.985. At 24 and 48 h, the total timestamp coefficients remain approximately 0.963 and 0.956, respectively, demonstrating pronounced daily and multi-day recurrence.

The high 24- and 48-h coefficients partially quantify the strong daily and multi-day seasonal recurrence that is present in the original irradiance measurements. The high 10-min ratio mainly reflects short-term continuity. These two forms of time dependence motivate using both recent irradiance lags and observations at the same target time from previous days in the forecast. Table 3 shows that the dataset contains a significant amount of rapid irradiance changes rather than just mild, clear-sky conditions. The 10-min ramp magnitude is defined as $R_{t}=\left|G_{t}-G_{t-1}\right|$. Approximately 11.92% of daylight intervals exhibit a 10-min absolute irradiance change greater than 50 W/m^2^, while 4.26% exceed 100 W/m^2^. More severe ramps are less frequent, with 1.23% and 0.38% daylight intervals exceeding 200 and 300 W/m^2^, respectively. These events indicate short-term variability, likely associated with rapidly changing cloud conditions and therefore posing a challenging test for forecast models. The ramp analysis complements the aggregated RMSE and MAE results by showing that the dataset includes transient conditions where short-term prediction is particularly difficult.

### 2.7. Chronological Distribution Drift

The chronological division produces a meteorologically challenging final testing period. The test set spans November and December and is therefore colder and characterized by higher pressure than much of the training period. Table 4 reports the largest standardized mean differences between the chronological training and test periods. Atmospheric pressure and air temperature exhibit the strongest shifts, whereas the target mean changes relatively little, with a standardized mean difference in irradiance of approximately −0.054.

This pattern means that similar average irradiance levels can occur under different meteorological relationships. Therefore, the final holdout tests for temporal transfer rather than random interpolation within the same seasonal distribution. It also motivates expanding-window validation and the presentation of specific monthly reports. However, because the testing period covers only the final part of a year, the resulting performance should not be interpreted as evidence of geographic or year-to-year generalization.

### 2.8. Feature Engineering

Feature construction uses only information available at the forecast origin, along with known deterministic variables for the future target timestamp. No meteorological or irradiance variable measured at the target time is included as input. All fitted preprocessing parameters are estimated from the corresponding training fold. The purpose of feature engineering is to expose complementary sources of predictive information that are not adequately represented by raw snapshot measurements alone. In particular, the engineered features represent the current atmospheric state, short-term irradiance persistence, recent variability and trends, daily and annual periodicity, and recurring conditions at the same target time on previous days. These information sources have different importance at different forecast horizons. Recent irradiance is expected to be particularly informative over very short horizons, whereas seasonal recurrence becomes increasingly relevant as forecast lead time increases. Meteorological variables provide additional information about local atmospheric conditions that can help learning algorithms model deviations from recent and recurrent irradiance patterns. Feature engineering, therefore, gives tabular models an explicit representation of the temporal and meteorological structure of the forecasting problem rather than requiring them to infer all temporal relationships from instantaneous measurements.

Sensor families include wind speed measurements from the upper and lower anemometers, wind direction measurements from the upper and lower vanes, relative humidity, air temperature, and atmospheric pressure. Current and historical irradiance measurements are also included when the selected feature profile allows irradiance history. The average measurements constitute the core predictors because they represent the core conditions observed during each 10-min interval. Selected minimum, maximum, and standard deviation statistics may also be retained when they have a clear physical interpretation and do not duplicate information contained in excluded anonymous or proxy channels. The wind direction is circular; $0^{\circ }$ and $360^{\circ }$ represent the same direction, although their gross numerical difference is large. Equation (7) represents each observation of wind direction $\theta_{t}$.

(7) $$w_{t}^{\sin}=\sin\;\left(\frac{\pi \theta_{t}}{180}\right),\;w_{t}^{\cos}=\cos\;\left(\frac{\pi \theta_{t}}{180}\right).$$

The raw angular variable is removed from the default feature configuration after these components are generated. Calendar features are created for both the forecast origin *t* and the deterministic target time t+h. The periodic components of the minutes of the day are defined as

(8) $$s_{t}^{\mathrm{minute}}=\sin\;\left(2\pi \frac{m_{t}}{1440}\right),\;c_{t}^{\mathrm{minute}}=\cos\;\left(2\pi \frac{m_{t}}{1440}\right),$$

and the annual components are

(9) $$s_{t}^{\mathrm{doy}}=\sin\;\left(2\pi \frac{d_{t}-1}{365.25}\right),\;c_{t}^{\mathrm{doy}}=\cos\;\left(2\pi \frac{d_{t}-1}{365.25}\right),$$

where $m_{t}$ indicates the minute of the day and $d_{t}$ indicates the day of the year. The Equations (8) and (9) avoid the discontinuities associated with representing calendar periodic variables using ordinary linear values. The lagged variables represent recent dynamics, intraday evolution, and daily recurrence. Equation (10) defines the lag feature for a source variable $x_{t}$.

(10) $$x_{t}^{\left(\ell \right)}=x_{t-\ell },$$

where *ℓ* is the lag expressed in 10-min sampling steps. For a historical window containing *L* observations, the rolling mean is

(11) $${\bar{x}}_{t,L}=\frac{1}{L}\sum_{j=0}^{L-1}x_{t-j},$$

while the endpoint slope is

(12) $$s_{t,L}=\frac{x_{t}-x_{t-L+1}}{L-1}.$$

Equations (11) and (12) summarize the recent level and trend of a historical predictor without using future observations. All rolling features are calculated using historical samples only. A rolling feature is considered valid only when the entire window is available, preventing partial or gap-spanning windows from introducing inconsistent temporal summaries. Seasonal analogs are aligned with the timestamp of the future target rather than the forecast origin. Let τ=t+h be the target time, with time indices counted in 10-min sampling steps (so t+h is equivalent to t+hΔ, where Δ=10 min), and let D=144 be a day in 10-min steps. Equation (13) defines the seasonal analog of a day.

(13) $$S_{\tau }^{\left(1\right)}=G_{\tau -D},$$

Equation (14) defines the two-day seasonal.

(14) $$S_{\tau }^{\mathrm{mean}2}=\frac{G_{\tau -D}+G_{\tau -2D}}{2},$$

and Equation (15) defines the seven-day seasonal median,

(15) $$S_{\tau }^{\mathrm{med}7}=\mathrm{median}\left\{G_{\tau -D},G_{\tau -2D},\ldots ,G_{\tau -7D}\right\}.$$

These seasonal features follow the use of daily and multi-day references in solar forecasting [17,18]. A seasonal analog is used only when its source timestamp is available no later than the forecast origin after taking into account the configured observation availability delay. Therefore, these variables are historical target measurements, not future observations. The learned models are trained to predict a seasonal baseline correction of the one-day seasonal irradiance baseline. The residual target is defined as

(16) $$r_{t+h}=G_{t+h}-G_{t+h-144},$$

and the corresponding absolute irradiance forecast is reconstructed as

(17) $${\hat{G}}_{t+h}=G_{t+h-144}+{\hat{r}}_{t+h}.$$

Thus, Equation (16) removes the one-day seasonal reference during model fitting, while Equation (17) converts the predicted residual back to absolute irradiance units before evaluation.

### 2.9. Feature-Profile Ablations

Three main feature profiles are defined to determine the sources of forecast skill. The irradiance history-only profile contains current and historical irradiance measurements, lagged irradiance values, rolling irradiance statistics, deterministic calendar variables, and permitted seasonal analogs. This setup evaluates how effectively future irradiance can be predicted from its temporal structure alone, without using meteorological measurements. The meteorological-only profile contains the named variables of wind speed and direction, humidity, temperature, and atmospheric pressure, along with deterministic calendar features. All solar sensor measurements and variables derived from the target are excluded from this profile. Therefore, it measures forecast information based on local meteorological conditions, regardless of current or historical irradiance. The irradiance-plus-meteorological profile corresponds to the default feature configurations. It combines the mentioned meteorological measurements with the explicitly allowed irradiance history, lag, rolling, calendar, and seasonal features. This profile evaluates whether meteorological variables provide complementary information beyond the strong temporal structure of the irradiance itself.

The three profiles are run as separate experiments to prevent target-derived information from being implicitly introduced into a meteorological-only analysis. Electrical and anonymous channels are reserved for complementary experiments unless the physical meanings, measurement time, and operational availability of V, I, T, D1, and D2 are independently verified. This separation preserves a clear distinction between forecasts based on environmental and irradiance observations and forecasts based on the broader state of the power system.

## 3. Forecasting Models and Experimental Methodology

**Figure 3:**
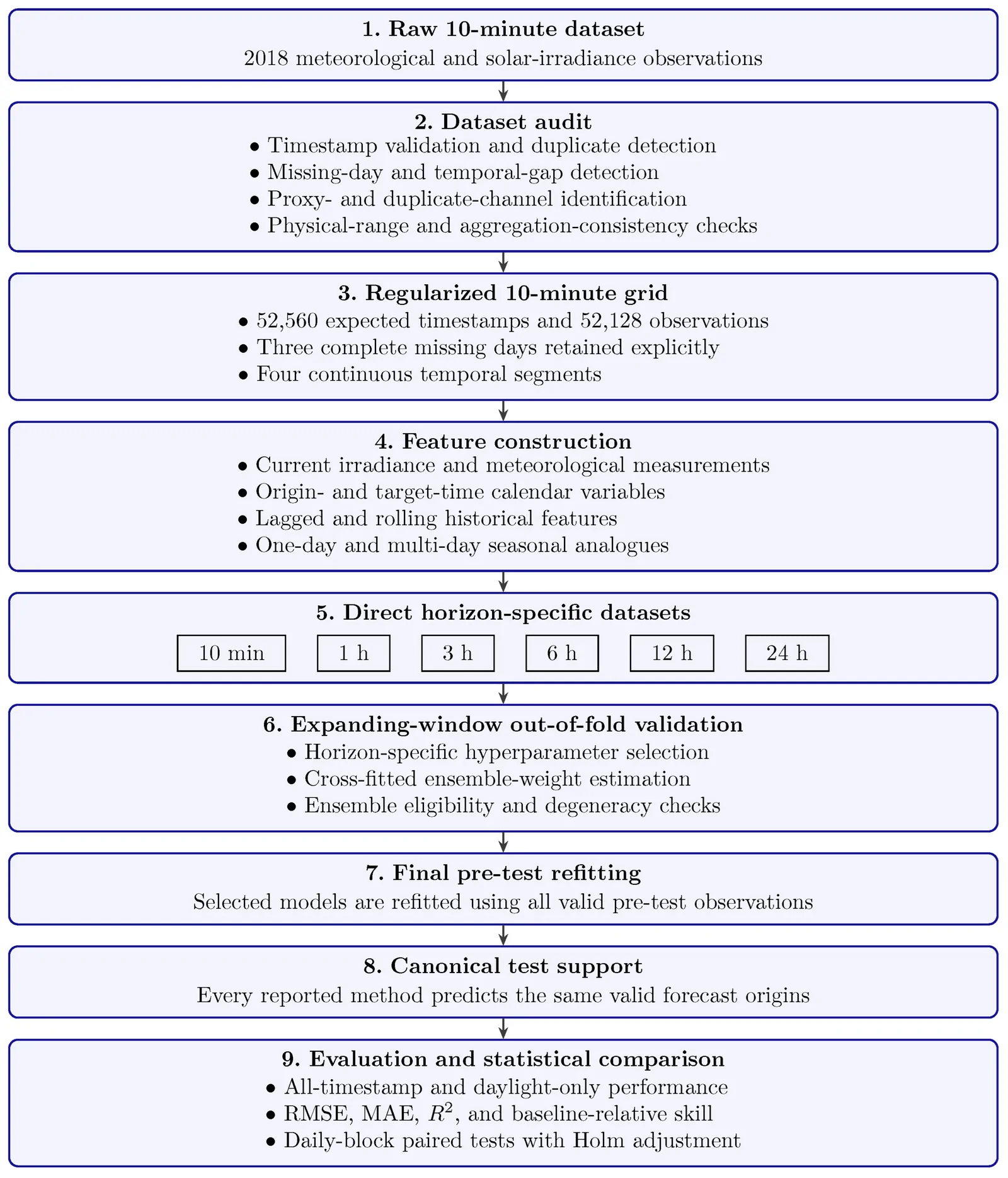
Framework for multi-horizon solar irradiance forecasting. The workflow comprises dataset auditing, gap-preserving temporal regularization, feature construction, direct horizon-specific dataset generation, expanding-window out-of-fold model selection, and final pre-test refitting, common-support testing, and daily-block statistical comparison.

This section presents forecast formulation, reference models, learning algorithms, convex forecast combinations, chronological validation protocol, evaluation support, statistical analysis, and reproducibility procedures. The methodology was designed to avoid temporal leakage and ensure that each headline comparison is performed on the same forecast origins. Figure 3 summarizes the complete experimental workflow adopted in this study. The process begins with raw 10-min meteorological and irradiance observations, which are first subjected to a systematic dataset audit. This audit verifies the integrity of the timestamp, identifies the three complete missing-day events, detects duplicate channels or target proxies, and verifies the physical and statistical consistency of the recorded sensor variables. Observations are then mapped onto a full 10-min temporal grid, while missing days are retained as explicit slots dividing the annual record into four continuous segments. After regularization, leakage-safe predictors are constructed from currently available sensor measurements, deterministic forecast-origin and target-time calendar variables, historical lags, rolling statistics, and seasonal target-time analogs. A separate direct forecasting dataset is formed for each of the six lead times: 10 min, 1 h, 3 h, 6 h, 12 h, and 24 h. Model configurations and convex ensemble weights are selected using chronologically ordered expanding-window out-of-fold predictions, ensuring that future validation and test observations do not influence model development. After model selection, each estimator is refitted using all valid pre-test observations. The final forecasts are restricted to a predeclared canonical test support, so that each reported method is evaluated on the same forecast origins valid at a given horizon. Performance is evaluated for both the full test support and the daylight-only subset using metrics of absolute error, coefficient of determination, and skill relative to strong persistence and seasonal references. Paired daily block tests with Holm adjustment are also used to distinguish numerical improvements from statistically supported gains. Therefore, the framework integrates data quality control, temporal leakage prevention, fair comparison of common support, and horizon-specific model selection within a single reproducible forecasting process.

### 3.1. Direct Multi-Horizon Forecasting Problem

Let $G_{t}$ denote the average solar irradiance measured at the forecast origin *t*, and let $x_{t}$ contain all predictors that are available when the forecast is given. Since the dataset is sampled at 10-min intervals, the sampling interval Δ is defined in Equation (18).

(18) Δ=10min.

The forecast horizon *h* is expressed as an integer number of sampling steps. Accordingly, Equation (19) defines the target time τ associated with forecast origin *t* and horizon *h*.

(19) τ=t+hΔ.

A separate direct forecasting function is trained for each forecast horizon. Using the target-time definition in Equation (19), Equation (20) expresses the horizon-specific forecasting problem.

(20) $${\hat{G}}_{t+h\Delta }=f_{h}\left(x_{t}\right).$$

Here, ${\hat{G}}_{t+h\Delta }$ denotes the forecast of solar irradiance at the target time t+hΔ, while $f_{h}\left(\cdot \right)$ denotes the forecasting function trained specifically for horizon *h*. The predictor vector $x_{t}$ contains only information available at the forecast origin *t*. Equation (21) defines the set of forecast horizons evaluated in this study.

(21) H={1,6,18,36,72,144}.

Because each element of H in Equation (21) represents several 10-min sampling intervals defined by Equation (18), these horizons correspond to lead times of 10 min, 1 h, 3 h, 6 h, 12 h, and 24 h, respectively. Chronological split membership for model fitting and validation is determined using the target time defined in Equation (19). Thus, a forecast origin is not assigned to a training period when its corresponding target occurs in a later validation period. This target-time boundary rule prevents forecast targets from crossing chronological model-selection boundaries.

### 3.2. Baseline Methods

Robust baseline methods are needed because ordinary persistence weakens with longer lead times, while daily recurrence remains highly informative for the audited dataset. Four deterministic reference models were included. Persistence and seasonal persistence are widely used reference models in solar forecasting, although the choice of the appropriate reference depends on the forecast horizon and the temporal structure of the data [5,17,18]. Ordinary persistence assumes that the most recent available irradiance remains unchanged, as shown by Equation (22),

(22) $${\hat{G}}_{t+h}^{P}=G_{t}.$$

#### 3.2.1. Seasonal Persistence

Seasonal persistence predicts future irradiance using the one-day seasonal analogue defined in Equation (13). Accordingly, the baseline forecast is set to ${\hat{G}}_{\tau }^{\mathrm{SP}}=S_{\tau }^{\left(1\right)}$. Since D=144, this corresponds to the irradiance observed at the same target clock time one day earlier. The source timestamp must be observable at the forecast origin after accounting for the configured observation availability delay.

#### 3.2.2. SeasonalMean2Day

The SeasonalMean2Day baseline uses the two-day seasonal mean defined in Equation (14). Accordingly, the forecast is set to ${\hat{G}}_{\tau }^{\mathrm{SM}2}=S_{\tau }^{\mathrm{mean}2}$. Averaging the corresponding observations from the preceding two days reduces sensitivity to an anomalous preceding day.

#### 3.2.3. SeasonalMedian7Day

The SeasonalMedian7Day baseline uses the seven-day seasonal median defined in Equation (15). Accordingly, the forecast is set to ${\hat{G}}_{\tau }^{S7M}=S_{\tau }^{\mathrm{med}7}$. This reference is more robust to isolated abnormal days, although it may respond more slowly to persistent weather changes.

When all horizons are evaluated at a common set of target timestamps, seasonal persistence can produce the same error at different horizons. This is expected and not an implementation defect. For a fixed target time τ, both the true value $G_{\tau }$ and the reference value $G_{\tau -144}$ remain unchanged; only the origin of the corresponding forecast t=τ−h differs.

### 3.3. Individual Learning Models

The comparison emphasizes boosted-tree regressors while retaining linear and neural tabular models as secondary references.

#### 3.3.1. Linear and Neural Tabular Models

The Ridge estimator in Equation (23) provides a regularized linear reference and is particularly useful when engineered lag and rolling features are strongly correlated [33]. Ridge regression minimizes a squared-error objective with $\ell_{2}$ regularization:

(23) $$\hat{\beta }=\arg\min_{\beta }\left[\sum_{i=1}^{N}{\left(y_{i}-\beta_{0}-x_{i}^{⊤}\beta \right)}^{2}+\alpha {∥\beta ∥}_{2}^{2}\right].$$

A multilayer perceptron (MLP) is included as a nonlinear tabular neural-network comparator. It uses standardized, median-imputed inputs, nonlinear hidden layers, regularization, and early stopping. It is not assumed to be superior; instead, it tests whether a generic feedforward neural model can exploit the engineered feature representation [34].

#### 3.3.2. Boosted-Tree Models

Four implementations of gradient boosting form the main family of learning models. HistGradientBoosting provides a histogram-based implementation of gradient boosted regression trees. XGBoost combines regularized boosting, shrinking, feature subsampling, and efficient tree construction [30]. LightGBM also uses histogram-based learning, but applies leaf-wise tree growth to improve the computational efficiency and flexibility of the model [31]. CatBoost employs additional regularization and ordered boosting mechanisms to reduce prediction shift and improve generalization [32]. These models are appropriate for the present dataset because the predictors are structured, the sample size is moderate, and the temporal information is represented through explicit lag, rolling, calendar, and seasonal features. Boosted trees can capture nonlinear relationships and interactions between these predictors without requiring input scaling.

The boosted-tree hyperparameters were selected independently for each forecast horizon using the same predefined candidate set and chronological validation protocol. Four expanding-window validation folds were used. Each validation block covered 30 days, and at least 120 days of preceding observations were required before the first validation block. For every candidate configuration, the model was fitted using only observations preceding the corresponding validation block. Predictions from all four validation folds were concatenated, and the configuration with the lowest out-of-fold RMSE was selected. This procedure was repeated independently for all six forecast horizons, allowing the selected hyperparameters to adapt to the information available at each horizon while keeping the search space and selection criterion identical. The final test period was not used during optimization. After selection, the chosen configuration was refitted using all valid pre-test observations and evaluated once on the untouched canonical test support. For XGBoost, Candidate 1 used 1000 trees, a learning rate (η) of 0.03, maximum depth 6, minimum child weight 2, row subsampling of 0.85, feature subsampling of 0.85, $L_{1}$ regularization of 0, and $L_{2}$ regularization of 1. Candidate 2 used 800 trees, a learning rate of 0.05, maximum depth 8, minimum child weight 3, row and feature subsampling of 0.80, $L_{1}$ regularization of 0.05, and $L_{2}$ regularization of 2. Both configurations used squared-error regression and histogram-based tree construction. For CatBoost, Candidate 1 used 1000 boosting iterations, a learning rate of 0.04, depth 7, $L_{2}$ leaf regularization of 3, and random strength of 1.0. Candidate 2 used 800 iterations, a learning rate of 0.05, depth 9, $L_{2}$ leaf regularization of 5, and random strength of 0.5. Both configurations optimized RMSE. Using the same set of candidates and the same expanding-window selection procedure at each forecast horizon provides a consistent chronological basis for model selection. This should be interpreted as robustness to temporally ordered validation rather than a test of seed-independent stochastic stability.

### 3.4. Convex Weighted Ensembles

Forecast combination is a well-established approach to exploiting complementary errors across methods, and regularized linear combination has been applied successfully to solar and wind forecasting [22,39]. The present work instead constrains the weights to the probability simplex, following the non-negativity constraint introduced for stacked regressions and the optimal convex combination of reference forecasts [17]. The simplex constraint ensures that each weight is interpretable as a share of the combination and prevents extrapolation beyond the range of the member forecasts. For *M* member forecasts, the combined prediction is defined by Equation (24), subject to the simplex constraints in Equation (25). For a set of *M* member forecasts, the convex combination is defined as

(24) $${\hat{G}}_{t}^{\mathrm{ens}}=\sum_{m=1}^{M}w_{m}{\hat{G}}_{t,m},$$

subject to

(25) $$w_{m}\geq 0,\;\sum_{m=1}^{M}w_{m}=1.$$

Weights are selected exclusively from expanding-window out-of-fold (OOF) predictions. For two-member sets, the weight of the first member is searched over a deterministic grid from 0 to 1, while the second weight is set to 1−w. For sets containing more than two members, the squared-error objective is solved on the probability simplex using an exact active-set least-squares procedure. This formulation guarantees non-negative weights whose sum equals 1. Weights below a pre-declared activity threshold can be pruned, while a set that effectively collapses to a single active member is identified as degenerate. A combination may also be rejected when its OOF improvement does not exceed a predefined minimum threshold. Additionally, ensembles that produce the same effective member-weighting signature are treated as duplicates, so equivalent forecast combinations are not reported as distinct methods. The final test set is not used to select ensemble members or optimize their weights. After OOF weight estimation, each member is refitted using all valid pre-test observations, and the fixed weights derived from validation are applied to the corresponding canonical test predictions.

### 3.5. Chronological Validation

The complete regularized 2018 time grid defines nominal chronological boundaries at 70% and 85% of the annual grid. These boundaries occur at approximately 13 September 2018 at 12:00 and 7 November 2018 at 06:00, respectively. The latter boundary defines the beginning of the final test period. Under the active expanding-window validation scheme, the nominal 70% boundary does not delimit the model-selection folds. It is retained for dataset diagnostics and training-derived data-quality summaries. Hyperparameters and ensemble parameters are selected using four consecutive 30-day expanding-window validation blocks ending at the final test boundary. The validation blocks are approximately 10 July–9 August, 9 August–8 September, 8 September–8 October, and 8 October–7 November 2018. For each fold, the training interval begins at the start of the dataset and extends forward in time, while the validation block remains strictly after all targets used for fitting. The first retained fold requires at least 120 days of preceding training history. This protocol more closely represents deployment under temporal drift than randomly shuffled cross-validation [15,16]. The training and validation sets are determined using the target timestamp. For a forecast origin *t*, horizon *h*, and sampling interval Δ, the target time is τ=t+hΔ. A training origin preceding a validation boundary *b* is retained only when

(26) t+hΔ<b.

The condition in Equation (26) prevents a training origin near a boundary from having its target inside a later validation period. The target-time condition is used for the expanding training and validation folds and for the final pre-test refitting set. The final test set forecast origins satisfy $t\geq b_{\mathrm{test}}$. Thus, every target used for final model fitting occurs strictly before the test boundary, while the first test forecast is issued at or after that boundary. This creates strict temporal separation between the final fitting targets and the untouched test origins.

### 3.6. Leakage-Safe Preprocessing

Each fitted preprocessing operation is estimated independently within the training portion of the corresponding expanding-window fold. Columns containing only missing training values are removed, and constant columns are identified using only training observations. Remaining missing values are imputed using estimated medians from the same training period, and missing indicators can be added when configured. For Ridge regression and MLP, scale parameters are also estimated exclusively from the training rows. The resulting fitted transformation is then applied unchanged to the associated validation block. Hyperparameter selection is based solely on the OOF predictions produced by the temporally ordered validation folds. The measured variables at the target timestamp are not used as predictors, and sequence windows that cross any interval of missing days are rejected. Consequently, neither the fitted preprocessing parameters nor the selected model configuration can use information from future validations or test observations.

### 3.7. Final Model Refitting

After the best candidate configuration is independently selected for each forecast horizon based on the concatenated expanding-window OOF RMSE, the corresponding preprocessing pipeline is refitted to all valid pre-test observations. The selected estimator is then retrained once with the chosen horizon-specific hyperparameters using all valid targets available before the final test boundary. This refitted model generates predictions for the untouched test period only once; thereafter, predictions are restricted to the predefined canonical test support before the headline performance measures are calculated. The reported experiment uses a single fixed, reproducible seed for stochastic learning models. Deterministic baselines are stored using the same seed-based directory and output structure to maintain a uniform experimental format. Repeated-seed evaluation is reserved for the subsequent robustness analysis. Table 5 summarizes the predefined candidate configurations evaluated for XGBoost and CatBoost. The same candidate set was considered independently at each forecast horizon, while model selection was based on the lowest expanding-window out-of-fold RMSE. This design keeps the hyperparameter search procedure consistent across horizons while allowing the selected model complexity to adapt to the forecasting lead time. The final test set was not used during hyperparameter selection.

### 3.8. Canonical Test Support

A predeclared, model-list-independent test set is constructed before model training. A forecast origin is kept only when its future target is observed, all required seasonal references are available, and the configured historical context is fully contained within one of the observed continuous temporal segments. When seasonal-residual learning is used, the corresponding historical target reference must also be available. In addition, the forecast origin must belong to the final test period, defined by $t\geq b_{\mathrm{test}}$. Target-time boundary enforcement is applied to the preceding training and model-selection periods, whereas final test membership is determined from the forecast origin. Every reported method must provide predictions for the same set of forecast origins. A model that cannot generate predictions for the complete canonical test support is treated as incomplete rather than being evaluated on a smaller or potentially easier model-specific subset. Consequently, differences in the reported performance reflect forecasting accuracy rather than differences in test-sample availability.

### 3.9. Performance Measures

Forecast accuracy is evaluated using complementary absolute, squared-error, normalized, bias, and reference-relative measures. RMSE and MAE are widely used in solar forecasting because they retain the physical units of the forecast target, while skill scores quantify performance relative to an explicit reference model [5,17]. For *N* common-support observations, the root mean squared error is

(27) $$\mathrm{RMSE}=\sqrt{\frac{1}{N}\sum_{i=1}^{N}{\left(G_{i}-{\hat{G}}_{i}\right)}^{2}}.$$

The mean absolute error is

(28) $$\mathrm{MAE}=\frac{1}{N}\sum_{i=1}^{N}\left|G_{i}-{\hat{G}}_{i}\right|.$$

The coefficient of determination is

(29) $$R^{2}=1-\frac{\sum_{i=1}^{N}{\left(G_{i}-{\hat{G}}_{i}\right)}^{2}}{\sum_{i=1}^{N}{\left(G_{i}-\bar{G}\right)}^{2}}.$$

For a reference model *B*, the RMSE skill score is

(30) $${\mathrm{Skill}}_{B}=1-\frac{{\mathrm{RMSE}}_{\mathrm{model}}}{{\mathrm{RMSE}}_{B}}.$$

Equation (27) is used as the primary model-ranking criterion, whereas Equation (28) describes the typical absolute forecast error. Equation (29) measures the proportion of target variance explained by the predictions. Equation (30) quantifies improvement relative to the specified reference forecast.

### 3.10. Conditional Evaluation

Aggregate metrics can hide important operational differences. The headline results are therefore reported for both the complete canonical test support and a daylight-only subset. The daylight analysis assesses whether the principal rankings remain valid after removing nighttime zero values. The implementation also generates month-specific, hour-specific, irradiance-regime, ramp-event, and ramp-magnitude diagnostics for supplementary analysis. The ramp categories are defined by absolute 10-min irradiance changes of 0–50, 50–100, 100–200, 200–300, and at least 300 $W/m^{2}$.

As a common physical post-processing rule, negative irradiance forecasts are clipped to zero before calculating the metric. No upper irradiance ceiling is imposed. Nighttime predictions are not forcibly set to zero. In particular, the observed-target daylight criterion is used only to define the daylight-only evaluation subset after forecasts have been generated and is never used as a predictor or as an operational post-processing input.

### 3.11. Statistical Testing

For each model and forecast horizon, the common-support absolute errors are aggregated by calendar day to reduce the influence of serial dependence among adjacent 10-min observations. The daily mean absolute errors of each candidate–reference pair are then compared using both a paired *t*-test and a Wilcoxon signed-rank test. The paired *t*-test evaluates whether the mean daily error difference is significantly different from zero under a parametric assumption, whereas the Wilcoxon test provides a nonparametric comparison based on the signed ranks of the paired daily differences.

(31) $$\Delta {\mathrm{MAE}}_{\mathrm{day}}={\mathrm{MAE}}_{\mathrm{candidate},\mathrm{day}}-{\mathrm{MAE}}_{\mathrm{reference},\mathrm{day}},$$

Equation (31) defines the daily paired error difference so that a negative value favors the candidate model. Paired *t*-tests and Wilcoxon signed-rank tests are applied to the daily paired errors, while Holm’s sequential procedure is used to control the family-wise error rate across related comparisons [20]. These inferential tests evaluate differences in daily mean absolute error and do not constitute significance tests of the point-level RMSE differences used for the primary model ranking. Accordingly, statistical significance in the following results refers specifically to $\Delta {\mathrm{MAE}}_{\mathrm{day}}$, whereas RMSE differences are interpreted as numerical performance differences.

### 3.12. Reproducibility

Each experiment stores the complete resolved configuration together with the chronological split boundaries and expanding-window fold definitions. The saved outputs also include the selected raw and engineered feature lists, fitted preprocessing objects, trained model artifacts, hyperparameter configurations, validation scores, and out-of-fold predictions. For the final evaluation, both native-support and canonical-support test predictions are preserved. Forecast-combination experiments additionally store the ensemble members and their selected weights. All experiments were conducted using Python version 3.10.18. The machine-learning implementations used scikit-learn version 1.5.2, XGBoost version 3.1.1, LightGBM version 4.7.0, and CatBoost version 1.2.8. Data processing and numerical operations were performed using pandas version 2.3.2 and NumPy version 2.2.6, while statistical analyses were performed using SciPy version 1.13.1. All runs were executed on Ubuntu 24.04.3 LTS with an AMD Ryzen 7 2700 eight-core processor, 64 GB of system memory, and an NVIDIA GeForce RTX 3090 GPU with 24 GB VRAM.

## 4. Experimental Results

This section evaluates the forecasting methods at lead times ranging from 10 min to 24 h. All methods are compared on a predeclared canonical test support of valid forecast origins at each horizon. This common-support design prevents model-specific differences in missing observations, temporal gaps, or sequence-window availability from affecting the rankings.

### 4.1. Evaluation Support and Reporting Conventions

Table 6 reports the number of test origins retained at each horizon. The support decreases from 7883 observations at 10 min to 7740 at 24 h because the test interval is defined by the forecast origin, and the final *h* origins lack targets in the observed 2018 record. RMSE is used as the primary ranking criterion because it emphasizes large forecast deviations, while MAE describes the typical absolute error. Skill relative to seasonal persistence (SP) is calculated using Equation (30) with B=SP. Positive values indicate improvement over the one-day seasonal reference.

### 4.2. Baseline Behavior

Table 7 compares the principal persistence and seasonal references. Seasonal persistence remains near 19.8 W/m^2^ across horizons because it uses the irradiance measured at the same clock time on the preceding day. The two-day seasonal mean is the strongest stand-alone reference at every horizon. Ordinary persistence is outperformed by the seasonal references even at 10 min because its error contains a systematic diurnal-phase component. Carrying the current observation forward always lags the clear-sky envelope by one sampling interval. A seasonal reference shares the target’s solar geometry exactly, so its only source of error is day-to-day atmospheric differences, which are small during the November–December test period.

At the 24-h horizon, persistence and seasonal persistence are mathematically equivalent. For a target at t+144, both methods use $G_{t}$, so their identical performance is expected.

### 4.3. 10 Min Forecasting

Table 8 shows that the tree-based learners dominate the 10-min horizon. XGBoost achieves the lowest RMSE under both evaluation conditions: 10.7769 W/m^2^ for all timestamps and 16.0605 W/m^2^ during daylight. These values correspond to seasonal persistence skill scores of approximately 45.5%. Random Forest produces the lowest MAE in both settings, with 3.5007 W/m^2^ overall and 7.7167 W/m^2^ during daylight. The difference between the RMSE and MAE winners indicates that XGBoost better controls the largest forecast errors, whereas Random Forest gives slightly smaller typical absolute deviations. All six tree ensembles outperform the seasonal baselines, demonstrating that recent irradiance and meteorological observations contain strong predictive information at this horizon.

### 4.4. 1 H Forecasting

Table 9 presents the one-hour results. CatBoost achieves the lowest RMSE, reaching 16.0788 W/m^2^ overall and 23.9065 W/m^2^ during daylight, representing an 18.67% improvement over seasonal persistence. LightGBM, XGBoost, and HistGradientBoosting follow closely, while Random Forest retains the lowest MAE. The two-day seasonal mean remains a competitive benchmark, but the leading boosting methods reduce its all-timestamp RMSE by approximately 12%. Ridge and MLP deteriorate markedly relative to the tree models, suggesting that nonlinear partition-based learners are better suited to the interactions between recent irradiance, meteorological variables, and calendar features at the one-hour lead time.

### 4.5. 3 H Forecasting

Table 10 shows the transition from purely learned models to seasonally guided forecast combinations. The HGB–SM2 combination ranks first for every principal metric, with an all-timestamp RMSE of 17.1372 W/m^2^, an MAE of 7.5165 W/m^2^, and a daylight RMSE of 25.5608 W/m^2^. Its seasonal-persistence skill is 13.36%, and its RMSE is approximately 6.45% lower than that of the two-day seasonal mean alone. The remaining combinations also rank near the top, whereas stand-alone CatBoost and XGBoost no longer surpass seasonal persistence. This result indicates that the stable same-time daily pattern becomes increasingly important at three hours, while the learned component remains useful for correcting deviations from that pattern.

### 4.6. 6 H Forecasting

Table 11 reports the six-hour performance. The XGBoost–SM2 combination obtains the minimum RMSE, 18.1749 W/m^2^ overall and 27.0413 W/m^2^ during daylight. However, the seasonal mean (2-day) gives the lowest MAE in both evaluations. The RMSE reduction relative to the two-day mean is only about 0.88%, while the MAE of the combination is higher. Thus, the principal benefit of the combination is concentrated in a limited number of large-error cases rather than in a uniform reduction of typical errors. The results also show that most stand-alone machine-learning models have negative skill relative to seasonal persistence at this horizon, confirming the growing dominance of the diurnal seasonal structure.

### 4.7. 12 H Forecasting

Table 12 presents the twelve-hour results. The HGB–SM2 and XGBoost–SM2 combinations are effectively tied: their all-timestamp RMSE values are 17.7842 and 17.7848 W/m^2^, and their daylight RMSE values are 26.6267 and 26.6286 W/m^2^, respectively. The numerical differences are too small to support a practical distinction between them. Both combinations improve on seasonal persistence by approximately 10.3% and on the two-day seasonal mean by about 3.2%. Stand-alone Extra Trees remains marginally better than seasonal persistence, while the other stand-alone learned models have negative seasonal-persistence skill. This horizon, therefore, benefits primarily from combining a learned correction with a strong multi-day seasonal reference.

### 4.8. 24 H Forecasting

Table 13 summarizes the 24-h forecasts. The XGBoost–SM2 combination achieves the best overall and daylight results, with RMSE values of 17.6602 and 26.3202 W/m^2^, respectively. It improves on both persistence and seasonal persistence by 11.29%, because the two baselines are identical at a one-day horizon: each uses the irradiance observed at the forecast origin. The HGB–SM2 combination is a close second, followed by HistGradientBoosting and the combinations based on one-day seasonal persistence. The two-day seasonal mean remains a strong stand-alone predictor, while CatBoost, MLP, and Ridge fail to improve on the one-day reference. The result supports the use of multi-day analog information for day-ahead forecasting, supplemented by a moderate data-driven correction.

### 4.9. Daily-Block Statistical Comparison

Table 14 reports paired comparisons between the RMSE-leading method at each horizon and the principal references. The tests are applied to daily MAE values rather than individual 10-min observations, reducing the influence of overlapping forecasts. Holm adjustment controls the family-wise error rate within each horizon and reference family.

The 10-min XGBoost improvement is significant against all three references under both paired tests. At one hour, CatBoost is decisively better than ordinary persistence, although its daily-MAE advantage over the seasonal references is not significant after adjustment. The horizon-specific combinations at 3, 6, and 12 h are also better than ordinary persistence, but the daily-MAE tests do not establish superiority over the strongest seasonal reference. At 24 h, none of the daily MAE differences are significant. These findings distinguish a clear short-term gain from the more modest long-horizon RMSE improvements.

### 4.10. Cross-Horizon Skill Analysis

**Figure 4:**
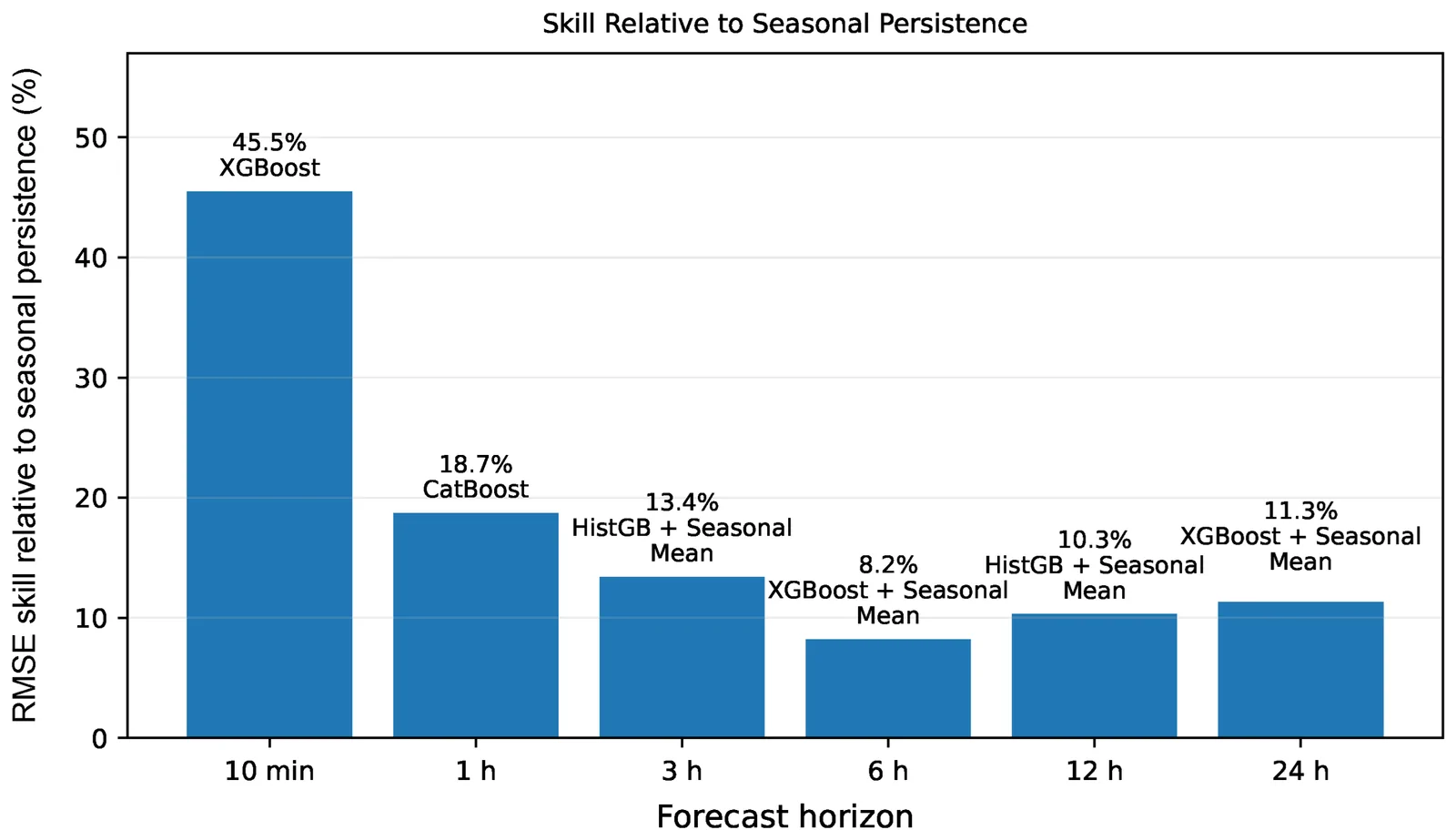
RMSE skill of the best-performing method at each forecast horizon relative to seasonal persistence. Positive values indicate a reduction in RMSE compared with the previous-day seasonal forecast. The model label above each bar identifies the best-performing method for the corresponding horizon.

Although the preceding tables provide detailed results for each forecast horizon, the relative benefit of the best-performing method changes considerably with forecast lead time. Figure 4 summarizes the RMSE skill of the best method at each horizon relative to seasonal persistence. The skill is calculated using Equation (30) with B=SP and is multiplied by 100 for presentation as a percentage.

The largest improvement was obtained for the 10-min horizon, where XGBoost achieved a skill of 45.5%. At the 1-h horizon, CatBoost reduced RMSE by 18.7% relative to seasonal persistence. For longer horizons, the best results were obtained from forecast combinations that incorporated the two-day seasonal mean. These methods achieved skill values of 13.4%, 8.2%, 10.3%, and 11.3% at the 3-, 6-, 12-, and 24-h horizons, respectively. The results demonstrate a transition in the dominant source of predictive information. Short-horizon forecasting benefits strongly from nonlinear learning of recent irradiance and meteorological conditions. As the forecast horizon increases, immediate observations become less informative, and forecasts increasingly benefit from daily seasonal structure. The positive skill observed at every horizon confirms that the selected methods consistently improve upon the previous-day seasonal persistence baseline, although the magnitude of improvement is greater for very-short-term forecasting.

### 4.11. Overall Interpretation

The results indicate a horizon-dependent change in the most effective forecasting strategy. At 10 min and 1 h, nonlinear tree-based learners effectively exploit recent irradiance and meteorological measurements. At three hours and beyond, the same-time multi-day irradiance pattern becomes the dominant source of predictive information, and the best results are obtained by combining this seasonal structure with a learned correction. The daylight-only rankings closely follow the all-timestamp rankings, showing that the conclusions are not produced solely by nighttime zero values. The high $R^{2}$ values reflect both accurate forecasts and the deterministic diurnal cycle of irradiance. For this reason, the interpretation emphasizes daylight errors and skill relative to seasonal persistence in addition to conventional aggregate metrics. Taken together, the results support XGBoost for very-short-term forecasting, CatBoost at the one-hour horizon, and seasonally guided boosting combinations for intraday and day-ahead prediction. The conclusions are limited by the use of data from a single site and a single year. After the three missing-day outages, the record comprises approximately 362 observed days, with the final test period concentrated in November and December. The meteorological variables exhibit measurable distribution drift between the training and test periods, and overlapping forecast origins are not independent weather events. Verified solar-geometry variables, numerical weather predictions, satellite imagery, sky-camera observations, and spatial cloud-motion information were not available.

Although the experimental results are site-specific, the proposed framework is transferable, at a methodological level, to other arid and tropical regions. The monitoring layer relies on commonly available meteorological measurements, including solar irradiance, wind speed, wind direction, air temperature, relative humidity, and atmospheric pressure. At a new site, the sensor configuration, mounting arrangement, sampling interval, and calibration should be selected according to local environmental conditions and measurement standards. The same computational workflow can then be applied, including timestamp and gap auditing, target-proxy screening, feature construction, chronological validation, horizon-specific model selection, and common-support evaluation.

The fitted models themselves, however, should not be transferred directly between climates without local validation and retraining. Preprocessing statistics, useful lag and rolling-window scales, seasonal analogs, and hyperparameters should be re-estimated from observations at the target site. Arid regions may exhibit strong clear-sky persistence but can differ in dust and aerosol loading, temperature extremes, and seasonal cloud occurrence. Tropical regions may exhibit stronger humidity, wet–dry seasonality, rapidly developing convective clouds, and greater short-term cloud variability. These differences can change the relative importance of recent irradiance, meteorological variables, and seasonal references. Verified solar-position and clear-sky variables may therefore be particularly useful when transferring the framework between locations. For multi-site deployment, the same audit and chronological evaluation protocol can be applied independently at each station or extended to a pooled multi-site learning design using explicit site metadata. Increasing the number of stations can additionally provide spatial information about cloud and weather evolution. For forecast horizons of several hours, further generalization is likely to benefit from numerical weather prediction, satellite cloud products, sky-camera observations, or spatial sensor networks. Consequently, validation on multiple years, stations, and climatic regions is required before the fitted models, or their ranking, can be regarded as geographically universal.

The current tables were generated from a single experimental seed to ensure exact reproducibility, but they do not quantify stochastic variation across repeated model fits. Models that involve randomized tree construction, subsampling, or stochastic optimization may produce slightly different error estimates under a different seed. This is particularly relevant when two methods have very similar RMSE or MAE values, since small stochastic changes could alter their numerical ordering. The optimized ensembles may also be affected indirectly because their weights are selected from the out-of-fold predictions of their member models. In contrast, deterministic persistence, seasonal-reference, and Ridge predictions are not affected by the random seed. Accordingly, the reported ranking should be interpreted as the ranking obtained under the reproducible seed-42 experiment rather than as evidence of seed-independent superiority. Repeated-seed experiments would provide a more complete assessment of ranking stability and stochastic uncertainty.

### 4.12. Comparison with Previous Studies

A direct numerical comparison with previous solar-forecasting studies requires caution because reported errors depend strongly on the site, climatic regime, target variable, sampling resolution, forecast horizon, daylight definition, input information, and train–test protocol. In the literature reviewed, no previous study was identified that used exactly the same 2018 Omdurman Ahlia University 10-min station record. We therefore compare the present findings with studies conducted under similar climatic conditions or using comparable short-term solar-irradiance forecasting settings.

A recent study by Adam et al. [41] estimated daily solar radiation in Gadarif, Sudan, using support vector regression, XGBoost, boosted regression forest, and k-nearest neighbors driven by daily temperature and a binary precipitation indicator. Their results showed that machine-learning methods can provide effective daily solar-irradiance estimates in a semi-arid Sudanese environment and that performance varies with local atmospheric conditions. Because that work targets daily totals rather than intraday forecast horizons, it is comparable to the present study in terms of setting rather than forecasting tasks. For high-resolution forecasting, Hategan et al. [22] evaluated intra-hour solar resource forecasts with lead times of 5 to 30 min using statistical methods, machine learning, and all-sky imagery. Similarly, Pena-Cruz et al. [42] investigated short-term DNI and GHI forecasting using ground-based sky imagery and cloud-motion information. Their reported GHI RMSE ranged from approximately 31.73 W/m^2^ at the 1-min forecast horizon to 75.02 W/m^2^ at the 10-min horizon. These values illustrate the strong dependence of absolute irradiance errors on the dataset and weather. The present 10-min RMSE is lower numerically, but this difference cannot be interpreted as direct model superiority because the two studies use different sites, meteorological conditions, sensing systems, input information, and test protocols. Previous studies and the present experiments both indicate that recent irradiance information and nonlinear learning are particularly valuable at very short lead times. The current study extends this comparison to longer horizons under one common protocol and shows that strong daily and multi-day seasonal references become increasingly competitive as the lead time grows. This finding also emphasizes why advanced models should be assessed against horizon-appropriate baselines rather than ordinary persistence alone.

## 5. Conclusions

This study investigated the time horizon-dependent forecast of solar irradiance using robust seasonal baselines, individual tabular learning models, and validation-selected convex combinations of tree models with seasonal references. The experimental framework was designed to reduce various sources of optimistic bias through auditing timestamps and sensor data, explicit preservation of time intervals, preprocessing exclusively for training, expanding-window model selection, exclusion of proxy channels from the target, and model-independent canonical test support. The results show that the most effective forecasting strategy varies with the time horizon. At 10 min, XGBoost achieved the lowest RMSE of 10.7769 $W/m^{2}$, while CatBoost achieved the lowest RMSE of 16.0788 $W/m^{2}$ at one hour. At 3, 6, 12, and 24 h, combinations of tree predictions boosted with the two-day seasonal mean achieved the lowest RMSE. These long-term improvements should be interpreted with caution. For example, at 6 h, the two-day seasonal mean maintained a lower MAE than the combination with the lowest RMSE, demonstrating that the preferred method may depend on the error criterion.

The pairwise significance analysis was based on mean daily absolute errors, rather than point RMSE. At 10 min, XGBoost achieved statistically significant reductions in daily MAE compared to persistence, seasonal persistence, and the two-day seasonal mean after Holm correction. Over longer horizons, the methods with the lowest RMSE generally did not establish statistically significant improvements in daily MAE relative to the strongest seasonal reference. Therefore, the results indicate that sophisticated learning models offer their greatest advantage over very short horizons. At the same time, the strong daily seasonal structure becomes increasingly difficult to improve as the forecast horizon increases. The use of a single season, a single annual cycle, and a single experimental seed limits the generalizability of the findings. Future work may evaluate the proposed framework for different years, locations, and climates, quantify stochastic variability through repeated seed experiments, and incorporate additional atmospheric information, such as numerical weather predictions, satellite observations, sky camera images, and verified solar geometry variables. Overall, the results support selecting specific models for each time horizon and demonstrate the importance of comparing machine learning-based predictions with seasonal references from the same forecast sources.

## Figures and Tables

**Table 1:** Main temporal properties of the irradiance dataset.

Property	Value
Observation period	January–December 2018
Nominal sampling interval	10 min
Observed timestamps	52,128
Expected regular-grid timestamps	52,560
Missing regular-grid timestamps	432
Timestamp completeness	99.178%
Invalid timestamps	0
Duplicate timestamps	0
Continuous temporal segments	4

**Table 2:** Selected lagged autocorrelation coefficients of the measured global solar irradiance. The coefficients were calculated directly from the non-deseasonalized irradiance series. The Daylight column uses the 06:00–18:00 local-clock screening interval employed only for preliminary descriptive analysis.

Lag	All Timestamps	Daylight
10 min	0.994	0.985
1 h	0.923	0.794
24 h	0.963	0.915
48 h	0.956	0.900

**Table 3:** Frequency of large absolute 10-min irradiance ramps during daylight.

Ramp Threshold	Daylight Proportion
$R_{t}>50\;W/m^{2}$	11.92%
$R_{t}>100\;W/m^{2}$	4.26%
$R_{t}>200\;W/m^{2}$	1.23%
$R_{t}>300\;W/m^{2}$	0.38%

**Table 4:** Largest standardized mean differences between the chronological training and test periods.

Variable	Standardized Mean Difference
Atmospheric pressure	1.147
Air temperature	−0.991
Relative humidity	−0.413
Bottom wind direction	−0.325
Solar irradiance target	approximately −0.054

**Table 5:** Candidate hyperparameter configurations used for XGBoost and CatBoost. The same candidate set was evaluated independently at each forecast horizon using expanding-window out-of-fold RMSE.

Model	Candidate 1	Candidate 2
XGBoost	1000 trees, η=0.03, depth =6 min. child weight =2	800 trees, η=0.05, depth =8 min. child weight =3
	subsample =0.85, column sample =0.85	subsample =0.80, column sample =0.80
	$L_{1}=0$, $L_{2}=1$	$L_{1}=0.05$, $L_{2}=2$
CatBoost	1000 iterations, η=0.04	800 iterations, η=0.05
	depth =7, $L_{2}=3$	depth =9, $L_{2}=5$
	random strength =1.0	random strength =0.5

**Table 6:** Canonical test support at each forecast horizon. The test-origin interval begins on 7 November 2018 at 06:00. For a horizon of *h* steps, the final *h* origins are removed because their targets fall beyond the end of the 2018 record. The daylight subset is defined by the observed target irradiance $G_{t+h}>20\;W/m^{2}$.

Horizon	Steps	All Timestamps	Daylight Only	Daily Blocks
10 min	1	7883	3546	55
1 h	6	7878	3541	55
3 h	18	7866	3529	55
6 h	36	7848	3511	55
12 h	72	7812	3481	55
24 h	144	7740	3481	54

**Table 7:** All-timestamp RMSE (W/m^2^) of the principal persistence and seasonal references.

Baseline	10 min	1 h	3 h	6 h	12 h	24 h
Persistence	21.9106	105.4961	284.5380	457.4821	520.0479	19.9084
Seasonal persistence	19.7640	19.7701	19.7794	19.7904	19.8164	19.9084
Seasonal mean (2-day)	18.3010	18.3066	18.3182	18.3354	18.3641	18.4493
Seasonal median (7-day)	19.3232	19.3287	19.3357	19.3457	19.3515	19.4413

**Table 8:** Forecasting performance for the 10 min horizon. Skill_SP_ is computed from all-timestamp RMSE relative to seasonal persistence.

Model	All Timestamps			Daylight Only			Skill_SP_ (%)
	RMSE	MAE	$R^{2}$	RMSE	MAE	$R^{2}$	
XGBoost	**10.7769**	3.6787	**0.9987**	**16.0605**	7.9968	**0.9957**	**45.47**
LightGBM	10.9095	3.6788	0.9986	16.2583	8.0401	0.9956	44.80
Extra Trees	11.1015	3.5439	0.9986	16.5485	7.8190	0.9954	43.83
HistGradientBoosting	11.1272	3.7179	0.9986	16.5832	8.1506	0.9954	43.70
Random Forest	11.1545	**3.5007**	0.9986	16.6263	**7.7167**	0.9954	43.56
CatBoost	11.1866	3.7789	0.9986	16.6585	8.0285	0.9954	43.40
Ridge	14.2358	6.6308	0.9977	19.6661	10.4915	0.9936	27.97
Seasonal mean (2-day)	18.3010	7.8556	0.9961	27.2760	17.3557	0.9876	7.40
MLP	18.7714	9.3238	0.9959	27.9092	19.3554	0.9870	5.02
Seasonal median (7-day)	19.3232	8.7548	0.9957	28.7992	19.3243	0.9862	2.23
Seasonal persistence	19.7640	8.0296	0.9955	29.4586	17.7449	0.9856	0.00
Persistence	21.9106	12.3170	0.9945	32.5258	26.7374	0.9824	−10.86

Note: HGB denotes HistGradientBoosting, SM2 denotes the two-day seasonal mean, and SP denotes seasonal persistence. Bold values indicate the best result in each column.

**Table 9:** Forecasting performance for the 1 h horizon. Skill_SP_ is computed from all-timestamp RMSE relative to seasonal persistence.

Model	All Timestamps			Daylight Only			Skill_SP_ (%)
	RMSE	MAE	$R^{2}$	RMSE	MAE	$R^{2}$	
CatBoost	**16.0788**	7.1901	**0.9970**	**23.9065**	14.9405	**0.9905**	**18.67**
LightGBM	16.1605	7.0204	**0.9970**	24.0738	15.1789	0.9903	18.26
XGBoost	16.3116	7.0126	0.9969	24.2928	15.1614	0.9902	17.49
HistGradientBoosting	16.4299	7.0836	0.9969	24.4833	15.1493	0.9900	16.90
Random Forest	16.8073	**6.6622**	0.9968	25.0568	**14.6750**	0.9895	14.99
Extra Trees	17.0155	7.0721	0.9967	25.3685	15.5940	0.9893	13.93
Seasonal mean (2-day)	18.3066	7.8585	0.9961	27.2950	17.3754	0.9876	7.40
Seasonal median (7-day)	19.3287	8.7569	0.9957	28.8187	19.3440	0.9862	2.23
Seasonal persistence	19.7701	8.0327	0.9955	29.4791	17.7656	0.9855	0.00
Ridge	29.0903	17.5222	0.9903	36.4113	25.2769	0.9779	−47.14
MLP	29.8475	14.8006	0.9898	44.2697	32.0716	0.9673	−50.97
Persistence	105.4961	65.9313	0.8721	153.6332	137.0364	0.6068	−433.62

Note: HGB denotes HistGradientBoosting, SM2 denotes the two-day seasonal mean, and SP denotes seasonal persistence. Bold values indicate the best result in each column.

**Table 10:** Forecasting performance for the 3 h horizon. Skill_SP_ is computed from all-timestamp RMSE relative to seasonal persistence.

Model	All Timestamps			Daylight Only			Skill_SP_ (%)
	RMSE	MAE	$R^{2}$	RMSE	MAE	$R^{2}$	
HGB–SM2 combination	**17.1372**	**7.5165**	**0.9966**	**25.5608**	**16.1122**	**0.9891**	**13.36**
XGBoost–SM2 combination	17.7755	7.9750	0.9964	26.5017	17.1509	0.9883	10.13
HGB–SP combination	18.0327	7.8758	0.9963	26.8928	16.7882	0.9880	8.83
Seasonal mean (2-day)	18.3182	7.8618	0.9961	27.3378	17.4153	0.9876	7.39
HistGradientBoosting	18.3360	8.4014	0.9961	27.3214	17.5931	0.9876	7.30
Random Forest	18.5248	7.9387	0.9961	27.6464	17.4444	0.9873	6.34
LightGBM	18.6258	8.5727	0.9960	27.7339	17.7639	0.9872	5.83
Extra Trees	18.7953	8.1191	0.9959	28.0507	17.9067	0.9869	4.98
XGBoost–SP combination	19.0588	8.6104	0.9958	28.4057	18.4265	0.9866	3.64
Seasonal median (7-day)	19.3357	8.7494	0.9957	28.8561	19.3632	0.9862	2.24
Seasonal persistence	19.7794	8.0332	0.9955	29.5206	17.7997	0.9855	0.00
CatBoost	19.8604	9.3875	0.9955	29.5921	20.1082	0.9854	−0.41
XGBoost	20.5952	9.7766	0.9951	30.6632	20.7135	0.9844	−4.12
MLP	26.7606	13.6730	0.9918	39.6501	27.4576	0.9739	−35.30
Ridge	66.3598	35.2627	0.9494	73.3724	44.0485	0.9105	−235.50
Persistence	284.5380	189.1076	0.0697	382.0933	335.6463	−1.4265	−1338.56

Note: HGB denotes HistGradientBoosting, SM2 denotes the two-day seasonal mean, and SP denotes seasonal persistence. Bold values indicate the best result in each column.

**Table 11:** Forecasting performance for the 6 h horizon. Skill_SP_ is computed from all-timestamp RMSE relative to seasonal persistence.

Model	All Timestamps			Daylight Only			Skill_SP_ (%)
	RMSE	MAE	$R^{2}$	RMSE	MAE	$R^{2}$	
XGBoost–SM2 combination	**18.1749**	8.4215	**0.9962**	**27.0413**	17.4902	**0.9878**	**8.16**
Seasonal mean (2-day)	18.3354	**7.8664**	0.9961	27.4021	**17.4744**	0.9875	7.35
HGB–SM2 combination	18.6651	9.0294	0.9960	27.6658	18.2503	0.9872	5.69
XGBoost–SP combination	19.1778	8.8908	0.9957	28.4969	18.2859	0.9865	3.10
LightGBM	19.2938	8.7919	0.9957	28.7718	18.7692	0.9862	2.51
Seasonal median (7-day)	19.3457	8.7392	0.9957	28.9118	19.3949	0.9861	2.25
Extra Trees	19.5446	8.4128	0.9956	29.2099	18.5854	0.9858	1.24
Seasonal persistence	19.7904	8.0258	0.9955	29.5787	17.8334	0.9854	0.00
HGB–SP combination	19.9420	9.8314	0.9954	29.4998	19.6875	0.9855	−0.77
XGBoost	20.3816	10.0537	0.9952	30.1623	20.2937	0.9848	−2.99
Random Forest	20.5210	9.0386	0.9951	30.6711	19.9848	0.9843	−3.69
HistGradientBoosting	21.4827	11.1755	0.9947	31.6037	21.9549	0.9833	−8.55
CatBoost	23.5003	11.5922	0.9936	35.0921	25.1336	0.9795	−18.75
MLP	46.7680	24.5913	0.9747	69.8744	53.8410	0.9186	−136.32
Ridge	63.4428	39.0003	0.9534	74.6824	49.8968	0.9070	−220.57
Persistence	457.4821	341.8105	−1.4208	530.2167	471.4243	−3.6872	−2211.64

Note: HGB denotes HistGradientBoosting, SM2 denotes the two-day seasonal mean, and SP denotes seasonal persistence. Bold values indicate the best result in each column.

**Table 12:** Forecasting performance for the 12 h horizon. Skill_SP_ is computed from all-timestamp RMSE relative to seasonal persistence.

Model	All Timestamps			Daylight Only			Skill_SP_ (%)
	RMSE	MAE	$R^{2}$	RMSE	MAE	$R^{2}$	
HGB–SM2 combination	**17.7842**	**7.6938**	**0.9963**	**26.6267**	**16.8766**	**0.9882**	**10.26**
XGBoost–SM2 combination	17.7848	7.7152	**0.9963**	26.6286	16.9929	**0.9882**	10.25
Seasonal mean (2-day)	18.3641	7.8653	0.9961	27.4997	17.5422	0.9874	7.33
XGBoost–SP combination	18.8350	8.0627	0.9959	28.1990	17.6683	0.9867	4.95
HGB–SP combination	19.1020	8.2301	0.9958	28.5979	17.9541	0.9863	3.61
Seasonal median (7-day)	19.3515	8.7128	0.9957	28.9781	19.4136	0.9860	2.35
Extra Trees	19.6855	8.4727	0.9955	29.4799	18.8826	0.9855	0.66
Seasonal persistence	19.8164	8.0150	0.9954	29.6768	17.8811	0.9853	0.00
LightGBM	20.5027	9.4767	0.9951	30.6862	20.6662	0.9843	− 3.46
XGBoost	20.5087	9.5659	0.9951	30.6805	20.5896	0.9843	−3.49
HistGradientBoosting	20.8521	9.9327	0.9950	31.1867	21.1592	0.9838	−5.23
Random Forest	22.3179	9.8134	0.9942	33.4251	21.8460	0.9813	−12.62
CatBoost	26.5640	12.8092	0.9918	39.7559	27.8006	0.9736	−34.05
MLP	51.9359	24.9545	0.9687	77.6244	53.9503	0.8994	−162.08
Ridge	92.4180	50.5524	0.9009	98.0098	59.2984	0.8396	−366.37
Persistence	520.0479	440.9358	−2.1376	548.7107	491.1196	−4.0277	−2524.33

Note: HGB denotes HistGradientBoosting, SM2 denotes the two-day seasonal mean, and SP denotes seasonal persistence. Bold values indicate the best result in each column.

**Table 13:** Forecasting performance for the 24 h horizon. Skill_SP_ is computed from all-timestamp RMSE relative to seasonal persistence.

Model	All Timestamps			Daylight Only			Skill_SP_ (%)
	RMSE	MAE	$R^{2}$	RMSE	MAE	$R^{2}$	
XGBoost–SM2 combination	**17.6602**	**7.6287**	**0.9964**	**26.3202**	**16.7008**	**0.9884**	**11.29**
HGB–SM2 combination	17.7452	7.7738	**0.9964**	26.4460	16.9902	0.9883	10.87
HistGradientBoosting	18.3464	8.5795	0.9961	27.3005	18.1491	0.9876	7.85
XGBoost–SP combination	18.3703	7.8157	0.9961	27.3783	17.0486	0.9875	7.73
Seasonal mean (2-day)	18.4493	7.9383	0.9961	27.4997	17.5422	0.9874	7.33
HGB–SP combination	18.4619	7.9431	0.9961	27.5118	17.2673	0.9874	7.27
Random Forest	18.5638	8.1648	0.9960	27.6697	17.9387	0.9872	6.75
Extra Trees	18.6227	8.2985	0.9960	27.7538	18.3087	0.9871	6.46
XGBoost	18.9777	8.6812	0.9958	28.2650	18.6081	0.9867	4.67
LightGBM	19.2560	8.8997	0.9957	28.6886	19.4093	0.9863	3.28
Seasonal median (7-day)	19.4413	8.7936	0.9956	28.9781	19.4136	0.9860	2.35
Persistence	19.9084	8.0894	0.9954	29.6768	17.8811	0.9853	0.00
Seasonal persistence	19.9084	8.0894	0.9954	29.6768	17.8811	0.9853	0.00
CatBoost	20.8114	12.3982	0.9950	29.8777	20.7605	0.9851	−4.54
MLP	48.1192	24.6237	0.9732	71.7352	54.3845	0.9141	−141.70
Ridge	66.4250	48.0478	0.9490	74.1577	56.1391	0.9082	−233.65

Note: HGB denotes HistGradientBoosting, SM2 denotes the two-day seasonal mean, and SP denotes seasonal persistence. Bold values indicate the best result in each column.

**Table 14:** Daily-block MAE comparisons for the RMSE-leading method at each horizon. ΔMAE_day_ is candidate minus reference; negative values favor the candidate. The reported *p*-values are Holm-adjusted.

Horizon	Candidate	Reference	ΔMAE_day_	Paired-*t* $p_{H}$	Wilcoxon $p_{H}$
10 min	XGBoost	Persistence	−8.6631	$9.954\times 10^{-46}$	$1.329\times 10^{-9}$
		Seasonal mean (2-day)	−4.1652	$1.255\times 10^{-11}$	$1.329\times 10^{-9}$
		Seasonal persistence	−4.3470	$3.977\times 10^{-11}$	$1.405\times 10^{-9}$
1 h	CatBoost	Persistence	−58.8435	$2.509\times 10^{-57}$	$1.329\times 10^{-9}$
		Seasonal mean (2-day)	−0.6519	0.5751	1.0000
		Seasonal persistence	−0.8345	0.1120	0.5152
3 h	HGB–SM2 combination	Persistence	−181.7939	$2.112\times 10^{-65}$	$1.772\times 10^{-9}$
		Seasonal mean (2-day)	−0.3412	1.0000	1.0000
		Seasonal persistence	−0.5212	0.7624	1.0000
6 h	XGBoost–SM2 combination	Persistence	−333.7390	$9.683\times 10^{-64}$	$1.772\times 10^{-9}$
		Seasonal mean (2-day)	0.5471	0.5972	0.4778
		Seasonal persistence	0.3802	0.9212	1.0000
12 h	HGB–SM2 combination	Persistence	−433.2723	$1.885\times 10^{-64}$	$1.772\times 10^{-9}$
		Seasonal mean (2-day)	−0.1570	1.0000	1.0000
		Seasonal persistence	−0.3204	1.0000	1.0000
24 h	XGBoost–SM2 combination	Persistence	−0.4625	1.0000	1.0000
		Seasonal mean (2-day)	−0.3082	0.2420	0.1716
		Seasonal persistence	−0.4625	1.0000	1.0000

## Data Availability

The original contributions presented in this study are included in the article. Further inquiries can be directed to the author.
